# Unique spatially and temporary-regulated/sex-specific expression of a long ncRNA, *Nb-1*, suggesting its pleiotropic functions associated with honey bee lifecycle

**DOI:** 10.1038/s41598-024-59494-6

**Published:** 2024-04-15

**Authors:** Hiroto Tadano, Hiroki Kohno, Hideaki Takeuchi, Takeo Kubo

**Affiliations:** 1 Department of Biological Sciences, Graduate School of Science, The University of Tokyo, Bunkyo-ku, Tokyo 113-0033 Japan; 2 Present Address: Department of Integrative Life Sciences, Graduate School of Life Sciences, Tohoku University, Sendai, Miyagi 980-8577 Japan

**Keywords:** Long non-coding RNAs, Entomology

## Abstract

Honey bees are social insects, and each colony member has unique morphological and physiological traits associated with their social tasks. Previously, we identified a long non-coding RNA from honey bees, termed *Nb-1*, whose expression in the brain decreases associated with the age-polyethism of workers and is detected in some neurosecretory cells and octopaminergic neurons, suggesting its role in the regulation of worker labor transition. Herein, we investigated its spatially and temporary-regulated/sex-specific expression. *Nb-1* was expressed as an abundant maternal RNA during oogenesis and embryogenesis in both sexes. In addition, *Nb-1* was expressed preferentially in the proliferating neuroblasts of the mushroom bodies (a higher-order center of the insect brain) in the pupal brains, suggesting its role in embryogenesis and mushroom body development. On the contrary, *Nb-1* was expressed in a drone-specific manner in the pupal and adult retina, suggesting its role in the drone visual development and/or sense. Subcellular localization of *Nb-1* in the brain during development differed depending on the cell type. Considering that *Nb-1* is conserved only in Apidae, our findings suggest that *Nb-1* potentially has pleiotropic functions in the expression of multiple developmental, behavioral, and physiological traits, which are closely associated with the honey bee lifecycle.

## Introduction

Over the past decade, thousands of long non-coding RNAs (lncRNAs), which are non-translatable RNAs longer than 200 nucleotides, have been identified in various animals^1–5^. lncRNAs play a key role in a wide spectrum of cell regulatory functions, such as differentiation, development, and various physiological responses, through gene regulation, and their functions are linked to cell type-specific expression and subcellular localization^1–5^. The ratio of ncRNAs in the genome tends to increase with the increasing complexity of eukaryotic organisms, suggesting the importance of lncRNAs in evolutionary changes in higher eukaryotic organisms^6–8^. Recent studies have proposed that some genus-specific lncRNAs contribute to the expression of behavioral, morphological, and physiological traits that are characteristic of animal species^9–11^.

The European honey bee (*Apis mellifera* L.) is a well-characterized social insect^12,13^ that has been used as a model for studying social insects^14^. Individuals that constitute honey bee colonies have unique morphological and physiological traits associated with their social tasks^12,13^. Female honey bees develop from fertilized (diploid) eggs and differentiate into two castes: workers (labor caste) and queens (reproductive caste)^12,13^. While queens have well-developed ovaries and are engaged in laying eggs in the colony, workers have inactive ovaries and perform various tasks to maintain colony activity in an age-dependent manner. Young workers (nurse bees) are engaged in in-hive tasks, such as nursing their broods, and older workers (foragers) forage for nectar and pollen outside the hives. In contrast, drones develop from unfertilized eggs, and adult drones are engaged only in reproduction. Adult drones mate with a virgin queen in the air during a ‘nuptial flight’^12,13^. Drones have compound eyes that are much larger than those of workers and well-developed visual systems, including the retinae of compound eyes and optic lobes (OLs, the visual center of the insect brain), which are thought to be important for finding and mating with a queen during the ‘nuptial flight’^12,15^.

The honey bee workers inform their nestmate of the memorized location of the food source using ‘dance communication’^12,13^ and have been used as a model for the study of advanced brain functions^14^. The mushroom bodies (MBs) are a higher-order center of memory, learning, and sensory integration of the insect brain^16–18^, and the MBs of aculeate species, including the honey bee, are more elaborate when compared with those of primitive hymenopteran species^19–21^. Furthermore, neural activity is induced in the worker honey bee MBs after foraging flights, suggesting a role in information processing during the foraging flights^22–25^. Extensive studies have analyzed the molecular and cellular mechanisms underlying the structures and functions of MBs in honey bees^26–31^. However, the mechanisms that regulate the genus-specific expression of multiple developmental, behavioral, and physiological traits, that are characteristic of the honey bees remain to be elucidated.

Previous studies have identified several lncRNAs in honey bees^32–36^, including *Nurse bee brain-selective gene-1* (*Nb-1)*^35^. Recent genome-wide transcriptome analyses have comprehensively identified many lncRNAs in honey bees^37–41^. We originally identified *Nb-1* as an RNA whose expression in the brain decreases depending on the age-polyethism of workers^35^. The full-length *Nb-1* cDNA sequence is 599 base and contains no open reading frame encoding a protein; thus, the gene product of *Nb-1* is presumed to be a lncRNA. Simultaneously, *Nb-1* had no similarity to any nucleotide sequences in other insect genome databases other than that of Japanese honey bees (*Apis cerana*), so *Nb-1* was assumed to be a lncRNA specific to some hymenopteran insect species close to the honey bee. In addition, in situ hybridization revealed that cells expressing *Nb-1* are clustered and distributed at various locations in the brain and overlap with populations of neurosecretory cells and octopaminergic neurons, suggesting a role for *Nb-1* in worker task transition mediated by neural modulation in these cells^35^.

Wang et al*.* (2021) recently reported that *Nb-1* is the most highly expressed lncRNA in the embryonic transcriptomes of both male and female honey bees, but that its expression increases throughout embryogenesis only in the male embryos, suggesting its role in sex differentiation^40^. As described above, several genus-specific lncRNAs are thought to contribute to the expression of behavioral, morphological, and physiological traits that are characteristic of animal species^9–11^. Thus, we hypothesized that if *Nb-1* is conserved in a restricted group of hymenopteran insects (i.e., the genus *Apis*), including honey bees, it may be involved in the regulation of not only age-polyethism but also the expression of various morphological, behavioral, and physiological traits characteristic of honey bees^35^. In the present study, to evaluate this hypothesis, we reexamined the conservation of *Nb-1* among hymenopteran insect species and performed detailed analyses of developmentary regulated expression and subcellular localization of *Nb-1* during various developmental stages in both sexes.

## Materials and methods

### Search for *Nb-1* sequences in hymenopteran insect species

*Nb-1* sequence of *Apis mellifera* was obtained from NCBI database (accession number: AB485674.1) and used in BLASTn search against the genome assemblies of hymenopteran insects using SequenceServer (https://sequenceserver.com/)^42^. The sequences with an E-value smaller than a default cutoff value of 1.00E-05 were included in the result.

### Animals and tissues

European honey bee (*Apis mellifera*) colonies were purchased from a local dealer (Kumagaya Honeybee Farm, Saitama, Japan) and maintained at the University of Tokyo (Hongo Campus, Tokyo, Japan). Open mated (multiple drone inseminated) queens were anesthetized on ice, abdomens removed, and ovaries dissected. The ovaries and other abdominal tissues were frozen at -80 ºC until use. Nurse bees were collected while feeding their brood and foragers were collected when they returned to the colony after foraging with pollen loads^43^. Female and male embryos were collected from the worker cells and drone cells, respectively. First instar larvae were collected within 1 day after the hatching. L5F larvae, prepupae (PP), and P1-P9 pupae were also collected. After hatching, the honey bee worker goes through five larval instars and the pupal stage. The fifth-instar can be subdivided into three distinct stages: L5F, L5S and PP^44^. At the beginning of the fifth instar, the larva lies at the bottom of its honeycomb cell in the same position as in the previous instars (L5F larvae). Capping of the cell with wax marks the beginning of the spinning stage, during which the larva spins a cocoon (L5S larvae). Eventually, the larva reaches an upright position with the head pointing toward the top of the cell and becomes a prepupa (PP). Approximately 2 days later, the pupal molt occurs, and the pupal stage lasts approximately 9 days (P1-P9). The L5F larvae, PP, and P1-P9 pupae of workers were collected from the hives, and their stages were determined by their external appearance, such as eye pigmentation^44,45^. Larvae, pupae, and adults were anesthetized on ice, heads were removed, and brains were dissected from the heads with fine tweezers under the binocular microscope. Dissected tissue samples were then immediately used or stored frozen at -80 ºC until use. The number of individuals used in each experiment is indicated in the legend for each figure.

### Chromogenic in situ hybridization analysis

Total RNA was extracted from each worker brain using TRIZOL Reagent (Invitrogen), treated with DNase I (Invitrogen), and then reverse-transcribed using SuperScript III (Invitrogen) with oligo (dT) primer. Reverse transcription (RT)-PCR was performed using Ex Taq (TaKaRa, Tokyo, Japan) according to the manufacturer’s instructions. The amplification was performed using gene-specific primers for *Nb-1* (5′-GCATTGTGGCTTAGGGTATAG-3′ and 5′-CCATTTTAGAGAGTAGGGCATCTCG-3′). PCR products were then subcloned with the pGEM-T Vector System (Promega, Madison, WI), and sequenced. Plasmid containing the fragment cDNA for *Nb-1* was re-amplified by PCR with M13 forward and reverse primers, and the PCR product was purified. The biotin-labeled sense or antisense riboprobes of *Nb-1* RNA were prepared by in vitro transcription with the Biotin RNA Labeling Mix (Roche, Nutley, NJ).

Chromogenic in situ hybridization was performed essentially as described previously^35^. Briefly, the whole brains dissected from the heads of the fifth instar larvae, pupae and adults, and whole bodies of the first instar larvae were fixed with 4% paraformaldehyde in phosphate-buffered saline (PBS) at 4 °C overnight, dehydrated in graded ethanol-Clear Plus (Falma Company) series, and embedded in paraffin. Paraffin-embedded brains were sectioned at 10-μm thickness. All sections were mounted on MAS-coated glass slides. Paraffin sections were deparaffinized in Clear Plus and rehydrated.

The ovary dissected from the abdomen of the queen was embedded in O.C.T compound (Sakura Finetek Japan, Tokyo, Japan) and was sectioned at 10-μm thickness. All sections were mounted on APS-coated glass slides. These frozen sections were fixed with 4% paraformaldehyde in 0.1 M sodium phosphate buffer (PB) at pH 7.4 for 2 h at 4 °C. The paraffin sections were partially digested with 10 μg/ml proteinase K in TE buffer (10 mM Tris–HCl buffer, pH 8.0, containing 1 mM EDTA) for 20 min at room temperature, fixed with 4% paraformaldehyde in 0.1 M sodium phosphate buffer (PB) at pH 7.4 for 15 min at 4 °C, and treated with 0.2 N HCl for 10 min. The sections were placed in 0.1 M triethanolamine-HCl buffer, pH 8.0, containing 0.1 M acetic acid anhydride for 10 min, and then washed with PB for 1 min at room temperature. After dehydration through a graded series of ethanol (70%, 80%, 90%, and 100%), the sections were hybridized overnight with DIG-labeled riboprobes of *Nb-1* RNA at 60 °C. Riboprobes were diluted with hybridization buffer (10 mM Tris–HCl buffer, pH 7.6 containing 50% formamide, 200 mg/ml tRNA, 1 × Denhardt’s solution [0.02% Ficoll, 0.02% polyvinylpyrrolidone, and 0.02% bovine serum albumin (BSA)], 10% dextrane sulfate, 600 mM NaCl, 0.25% sodium dodecyl sulfate, and 1 mM EDTA) and preincubated for 10 min at 85 °C. After hybridization at 60 °C overnight in a moist chamber, the sections were washed with 5 × saline sodium citrate (SSC) buffer and 2 × SSC containing 50% formamide at 60% for 30 min, treated with 10 mg/ml RNase A (Sigma-Aldrich, St Louis, MO) in TNE (10 mM Tris–HCl pH 7.5; 0.5 M NaCl; 1 mM EDTA) buffer by incubating for 30 min at 37 °C, followed by washing with TNE buffer at 37 °C for 10 min, and successively washed with 2 × SSC and 0.2 × SSC twice for 20 min at 60 °C. DIG-labeled riboprobes were detected immunocytochemically with alkaline phosphatase-conjugated anti-DIG antibody using the DIG Nucleic Acid Detection Kit (Roche). The signals were examined under the microscope (Olympus, Tokyo, Japan).

### Fluorescence in situ hybridization

Fluorescence in situ hybridization was performed essentially as described previously^35^. The frozen sections of queen’s ovary and paraffin sections of the brains were pretreated as described above. Then, the sections were hybridized overnight with biotin-labeled riboprobes of *Nb-1* RNA at 60 °C. Riboprobes were diluted with the hybridization buffer and preincubated for 10 min at 85 °C. After hybridization at 60 ºC overnight in a moist chamber, the sections were washed and treated with RNae A (Sigma-Aldrich). Biotin-labeled riboprobes were detected using TSA Plus Fluorescein System (PerkinElmer) according to the manufacturer’s instructions. After counterstaining with 30 mM 4′-6-diamidino-2-phenylindole (DAPI, Invitrogen) in TNT buffer (100 mM Tris–HCl pH 7.5; 150 mM NaCl; 0.05% Tween 20), the signals were examined under the fluorescence microscope (Carl Zeiss) or the confocal laser-scanning microscope (LSM 710, Carl Zeiss). The images were merged using Adobe Photoshop CS4.

### Double labeling with in situ hybridization and BrdU immunohistochemistry

Larvae and pupae were injected into the head with 1 μl of 25 mg/ml 5-bromo-2-deoxyuridine (BrdU, Sigma) dissolved in bee saline (130 mM NaCl, 6 mM KCl, 4 mM MgCl_2_, 5 mM CaCl_2_, 160 mM sucrose, 25 mM glucose, 10 mM HEPES (pH 7.0)) containing 0.01% neutral red, and subsequently kept in an incubator (33 °C) for 20–24 h.

Immediately after dissection, the brains were fixed overnight in 4% paraformaldehyde in PBS at 4 °C. Then, paraffin embedding and sectioning were performed as described above. After that, fluorescence in situ hybridization was performed as described above until the detection step using the TSA Plus Fluorescein System (PerkinElmer).

### BrdU immunohistochemistry

After the fluorescence in situ hybridization, sections were transferred to PBS, incubated in 2 M HCl for 30 min at room temperature and washed three times in PBS. After blocking reaction in PBS containing 1.0% BSA and 0.25% Tween 20, sections were incubated with mouse monoclonal anti-BrdU antibody (BD Bioscience San Jose, CA, USA) diluted to 2.5 μg/ml in PBS containing 1.0% BSA and 0.25% Tween 20 overnight at 4 °C. Control sections were incubated with mouse normal IgG (Chemicon Europe) instead of anti-BrdU antibody. Sections were then washed four times in PBS, incubated with Alexa 555-conjugated goat anti-mouse antibody (Invitrogen) diluted to 1:1000 in PBS containing 1.0% BSA and 0.25% Tween 20 for 2 h at room temperature and washed four times in PBS. After counterstaining with 30 mM DAPI in water, the signals were examined under the fluorescence microscope (Carl Zeiss). The images were merged using Adobe Photoshop CS4.

### Northern blotting analysis

For each sample, total RNA from 5 brains, from which retinae were removed, of each stage from L5F to adult stage were extracted using TRIZOL Reagent (Invitrogen). Total RNA were also extracted from female embryos, male embryos, queen’s ovary or abdomen. These sample RNA were stored at -80 °C until use. The DIG-labeled sense or antisense riboprobes of *Nb-1* RNA were prepared as described above.

Northern blotting analysis was performed according to a standard protocol using DIG-labeled riboprobes with slight modification. Sample RNA were subjected to denaturing urea-polyacrylamide gel (5.0% polyacrylamide, 8 M urea) electrophoresis, and transferred to a nylon membrane. The membranes were hybridized with the riboprobes of *Nb-1* RNA at 68 °C in the hybridization mixture consisting of 50% formamide, 10 × Denhardt’s solution (Wako), 20 mM sodium phosphate (pH 6.5), 750 mM sodium chloride, 75 mM sodium citrate, 0.5% sodium dodecyl sulfate, and 100 μg/ml Herring sperm DNA (Promega). After washing, chemiluminescent detection was performed using DIG Wash and Block Buffer Set (Roche), antibody against DIG conjugated to alkaline phosphatase (Roche), and CDP-Star (Roche). Band intensities were quantitatively analyzed by ImageJ software version 1.54f. (https://imagej.net/ij, National Institutes of Health, Bethesda, MD).

## Results

### Investigation of *Nb-1* sequence conservation in hymenopteran insect species

We have previously reported that the *Nb-1* sequence of the European honey bee (*A. mellifera*) is conserved in the Japanese honey bee (*A. cerana japonica*) but not in other insect species whose genome assemblies had already been available, such as the fruit fly (*Drosophila melanogaster*) and the parasitoid wasp (*Nasonia vitripennis*)^35^. As genome assemblies of over 160 hymenopteran species are available today^46^, we first examined the conservation of *Nb-1* sequences in hymenopteran insect species. A nucleotide BLAST search detected sequences with 100% query coverage and over 80% identity to the *Nb-1* sequence of *A. mellifera* in the species of the genus *Apis* (Table S1). In contrast, other species, including the closely related species of the genus *Bombus*, have remarkably low query coverage (less than 20%), indicating that the *Nb-1* sequence is not conserved in these species. This result indicates that only species of the genus *Apis* possess the *Nb-1* sequence, suggesting potential genus-specific functions of *Nb-1*.

### Developmentally regulated *Nb-1* expression in the developing worker brain

Our previous quantitative RT-PCR analysis showed that *Nb-1* expression in adult worker honey bee brains decreases in an age-dependent manner^35^. In addition, Wang et al. (2021) identified *Nb-1* as the most abundantly expressed lncRNA in both female and male embryos in the embryonic transcriptome of the honey bee^40^. These findings raised the possibility that *Nb-1* is more strongly expressed in the developing brains of the worker honey bees at the larval and pupal stages than in the adult brains. To clarify this possibility, we investigated *Nb-1* expression levels in the developing brains during the larval and pupal stages.

**Figure 1:**
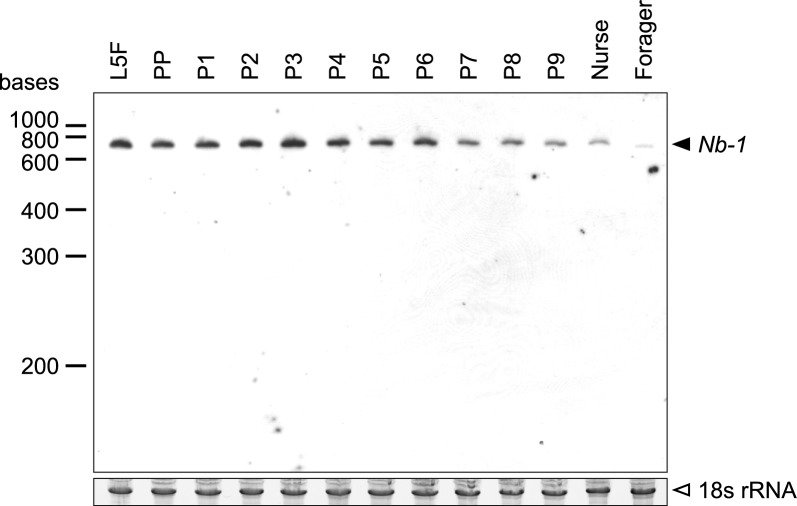
*Nb-1* expression in the larval, pupal, and adult worker brains. Northern blotting was performed using total RNAs extracted from the brains of workers at the five-instar larval feeding stage (L5F), prepupal stage (PP), pupal stages (P1–P9), and adult stages (nurse and forager) and antisense riboprobes for *Nb-1* RNA (upper panel). 5 individuals were used for each of the L5F, P1–P9, and Nurse bee samples, respectively, and 4 individuals were used for the Forager sample. The bands for 18S rRNA bands were also detected by staining with ethidium bromide as loading controls (lower panel). The bands corresponding to *Nb-1* RNA and 18S rRNA are shown with black and white arrowheads, respectively, on the right.

We performed Northern blotting analysis using total RNA extracted from the brains of fifth-instar larvae, pupae, and adults (nurse bees and forager bees) of worker honey bees. A single band of approximately 700 bases long, which corresponds to *Nb-1* RNA^34^, was detected in all samples tested (Fig. 1, Fig. S1). The intensities of the single bands gradually decreased from day 8 pupae to foragers (Fig. 1, Table S2). The band intensity of nurse bee brains was stronger than that of forager bee brains (Fig. 1, Table S2), which is consistent with our previous report^40^. These findings indicated that *Nb-1* expression gradually decreases in a developmental stage-dependent manner in the pupal and adult worker brains.

### Distribution of *Nb-1*-expressing cells in the developing worker brain

As *Nb-1* expression in developing pupal brains was stronger than that in adult brains (Fig. 1), we performed in situ hybridization of *Nb-1* RNA to investigate the distribution of *Nb-1*-expressing cells in the developing pupal brain.

The adult honey bee brain comprises several regions, such as the MBs, antenna lobes (ALs), a primary olfactory and mechanosensory center, OLs, a primary visual center, and the subesophageal ganglion (SOG), a center for tasting and feeding behavior^47^. In the honey bee, each MB comprises two cup-like calyces and a pedunculus, and consists of intrinsic interneurons, termed Kenyon cells, whose somata are located inside and on the outer surface of the calyces^48–50^. Adult AL neuropils are divided into spherical subcompartments, the olfactory glomeruli^47,51^, and adult OLs comprise three layers of nerve fibers: the lamina, medulla, and lobula, which are intercalated by layers of somata^47,52^. The development of MBs begins with two clusters of neuroblasts per hemisphere, located at the inner core of the inside of each developing calyx, at the early larval stage^53,54^. OLs comprise neuroblasts arranged in columns in the medullary layer at the fifth-instar larval stage^55^. At the fifth-instar larval stages, adult-like structures of OLs, ALs, and MBs already began to develop (Fig. S2A, F, K, P).

Using an antisense riboprobe against *Nb-1* RNA, we detected clear signals that were confined to a subpopulation of cells in the L5F and L5S brain regions (Fig. S2A,F,K,P); however, no signal was detected when the sense riboprobe was used (Fig. S2B, G, L, Q). *Nb-1*-expressing cell clusters were distributed in various locations in the brains of both L5F and L5S. *Nb-1* expression was strongly detected in the MB cell clusters located in the dorsal anterior part of the brain (Fig. S2D, O). *Nb-1*-expressing cell clusters also surrounded the spherical neuropils of the ALs (Fig. S2E, J, T). Moreover, *Nb-1* expression was detected in the outer parts of the OLs and inner parts of the retinae (Fig. S2C, H, M, R). In addition, other *Nb-1*-expressing cell clusters were distributed around the neuropils of the protocerebrum (Fig. S2E, I, N, S). This finding indicates that *Nb-1* is expressed in more cells in the brains of fifth-instar larvae than in adults^35^, which is consistent with our Northern blotting analysis results (Fig. 1).

The distribution of *Nb-1*-expressing cells in the brain of PP was similar to that in L5F (Fig. S3A, E). No signal was detected using the sense riboprobe (Fig. S3B, F). In developing MBs, two cell clusters with different *Nb-1* RNA signal intensities were detected. Cell clusters with strong *Nb-1* expression were located in the center of each calyx, whereas those with weak *Nb-1* expression were located on both sides of the clusters that strongly expressed *Nb-1* (Fig. S3C). In the ALs, many *Nb-1*-expressing cell clusters surrounded spherical neuropils (Fig. S3G). Moreover, cells with high *Nb-1* expression were located in the dorsal and ventral parts of the outer OL layer (Fig. S3H). Strong *Nb-1* RNA signals were also detected in the retinas (Fig. S3D).

During the pupal stage, Kenyon cell clusters continue to grow, but the proliferating neuroblasts are restricted to the most ventral and medial portions of each developing calyx in the MBs^54^. After the onset of apoptosis in MB neuroblasts, neuroblast proliferation abruptly ceases^46^, and proliferating neuroblasts completely disappear at the P6 stage^54^. In ALs, the neuropil differentiates into glomeruli after the pupal molt, and the completed ALs are organized into glomerulus-like structures by the P7 stage^55^. In OLs, many neuroblasts differentiate into neurons with increased neuropil extension at the P1 stage, and OLs are completely differentiated at the P4 stage^56^.

At the P1 stage, both cell clusters expressing *Nb-1* strongly in the center of the MB calyces and those expressing *Nb-1* weakly on both sides of the clusters located at the center of the calyces were detected (Figs. S4A, E, C, G), as in the PP. No staining was detected in the sections treated with the sense riboprobe (Figs. S4B, F, J). In the ALs, *Nb-1* expression was more restricted than in the larval brain (Fig. S4H). In the OLs, *Nb-1* signals were not as evident as those in the larvae and were restricted to regions lateral to the medulla, the second OL layer (Fig. S4L). Moreover, many *Nb-1*-expressing cells were located in the ventral and lateral regions of the SOG (Fig. S4K). These locations corresponded to those of the *Nb-1*-expressing cell clusters C8 and C5 observed in the adult brain^35^. In addition, many other *Nb-1*-expressing cell clusters were distributed around the neuropil of the protocerebrum, as observed in the PP (Fig. S4D).

At the P2 stage, the distribution pattern of *Nb-1*-expressing cells was similar to that at the P1 stage (Fig. S5). Two types of cell clusters with different *Nb-1* expression levels were detected in the MB calyces (Fig. S5B, G). *Nb-1*-expressing cell clusters were also detected in the ALs (Fig. S5D, I) and the regions surrounding the neuropil of the protocerebrum (Fig. S5C, H, L). Moreover, *Nb-1* expression was observed in the outer retinal layer (Fig. S5E, J, R). Furthermore, *Nb-1*-expressing cells were distributed around the SOG neuropils (Fig. S5M, N, Q). However, no signals for *Nb-1* were detected in the outer OL layer (Fig. S5O).

At the P3 stage, *Nb-1*-expressing cells were also detected in the developing MB calyces (Fig. S6C, H, M) and the surrounding neuropils of the ALs (Fig. S6D, I), protocerebrum (Fig. S6C, E, M), and SOG (Fig. S6N). However, the size of the *Nb-1*-expressing cell cluster located lateral to the AL neuropil was decreased (Fig. S6N). Moreover, a weak expression was detected in the retina (Fig. S6O). Only several *Nb-1*-expressing cells were observed in OLs (Fig. S6E).

At the P5 stage, three types of *Nb-1*-expressing cells with distinct signal intensities for *Nb-1* RNA were detected in the MBs (Fig. S7C, H, M). The cells with the strongest *Nb-1* expression were located in the center of the calyces, which corresponded to proliferating MB neuroblasts and ganglion mother cells^53^. The cells with the next strongest signal intensities surrounded the cell clusters with the strongest signal intensities, which may correspond to postmitotic neural cells. The cells with the weakest expression surrounded the cell layer with the second strongest staining and may correspond to maturing Kenyon cells. *Nb-1* was expressed in the cells surrounding the neuropil of the AL (Fig. S7E, J), protocerebrum (Fig. S7C, D, H, M), and SOG (Fig. S7O). Weak *Nb-1* expression was detected in the retinas (Fig. S7I). *Nb-1*-expressing cells were located between the lamina and retina and in the ventral region between the medulla and lamina (Fig. S7I). Several *Nb-1*-expressing cells were distributed in the posterior OL region (Fig. S7N).

At the P6 stage, *Nb-1* expression was observed in various regions surrounding the neuropils of the ALs (Fig. S8D, H), protocerebrum (Fig. S8B, C, F, G, J), and SOG (Fig. S8K, M). The expression pattern in the MBs, however, differed from that in the previous developmental stages. The expression was almost restricted to the center of the calyces and was weaker than that detected in other brain regions (Fig. S8B, F).

At the P7 stage, *Nb-1* expression was more restricted to a subpopulation of cells (Fig. S9). Weak *Nb-1* expression was detected in the center of the calyces of MBs as observed at the P6 stage (Fig. S9B, F, J). Cells expressing *Nb-1* surrounded the neuropils of the ALs (Fig. S9D and H) and SOG (Fig. S9K). The other *Nb-1*-expressing cells were located lateral to the medial calyces in the anterior section (Fig. S9C), at the dorsal anterior midline of the protocerebrum (Fig. S9F), lateral to the lateral calyces in the middle section (Fig. S9G), and posteriorly below the lateral calyces (Fig. S9J). *Nb-1* RNA signals were not detected in the OLs (Fig. S9L).

At the P8 stage, the *Nb-1* expression pattern in the brain is similar to that in adult worker honey bees^35^. In the MBs and OLs, no *Nb-1* expression was detected (Fig. S10C, H, M, O). *Nb-1*-expressing cells were detected anteriorly, below the lateral calyces, and in the dorsal anterior midline of the protocerebrum (Fig. S10C), which corresponded to *Nb-1*-expressing cell clusters C1 and C2 in the adult brain^35^. *Nb-1*-expressing cells were also located above the central complex, corresponding to C7 (Fig. S10H) between the OLs and the lateral calyx, corresponding to C6 (Fig. S10J), posteriorly below the lateral calyces, corresponding to C9 (Fig. S10M) and laterally and ventrally in the SOG, corresponding to C5 and C8 (Fig. S10N). Moreover, other *Nb-1*-expressing cells were visible surrounding the neuropils of the ALs (Figs. S10D, I) and lateral regions of the protocerebrum (Fig. S10E, J). Additionally, weak expression was still detected in the retina (Fig. S10O).

In summary, *Nb-1* expression spread more diversely in the brain at the late larval and early pupal stages than at the adult stage, including the developing MBs, and it decreased and disappeared gradually, becoming restricted to a small number of cells throughout the pupal stages. These findings were consistent with the Northern blotting results (Fig. 1) and suggested that the decrease in *Nb-1* expression in the whole brain during pupal development was mainly derived from a decrease in the number of *Nb-1*-expressing cells.

### *Nb-1* expression in the proliferating cells in the developing worker brain

Next, we focused on *Nb-1* expression in the developing MBs during the larval and early pupal stages. Our results indicated that strong *Nb-1* expression was detected in the center of the calyces up to the P5 stage, decreased during the P6/7 stages, and completely disappeared at the P8 stage (Fig. S11). Considering that proliferating MB cells are located in the center of the MB calyces in the pupal honey bee brains and that the proliferation ceased abruptly within 1 day after the onset of apoptosis in the proliferative clusters at the P4 stage^46,54^, our results raised the possibility that *Nb-1* is strongly expressed in the proliferating MB cells. To test this possibility, we injected 5′-bromo-2-deoxyuridine (BrdU), a thymidine analog that is incorporated in the DNA of proliferating cells during the S-phase of the cell cycle^57^, into the heads of larvae and pupae and performed double labeling with in situ hybridization for *Nb-1* RNA and immunohistochemistry for BrdU.

**Figure 2:**
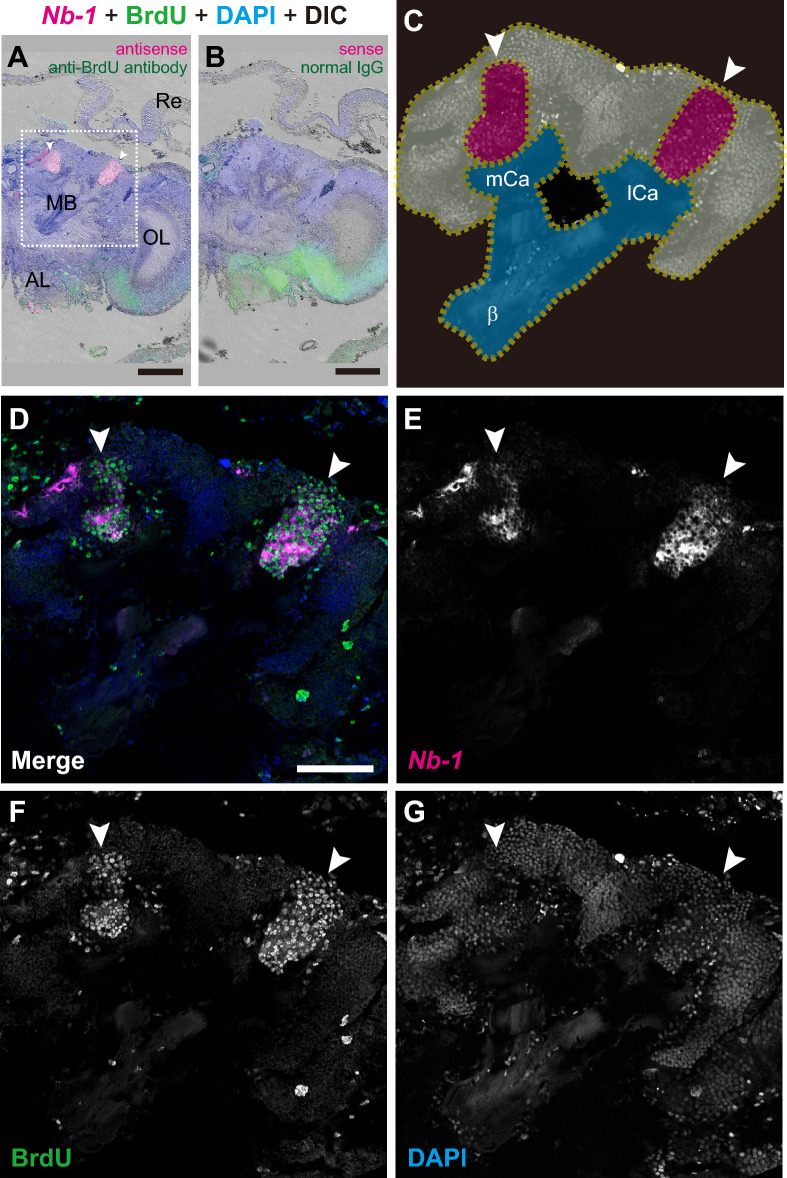
Distribution of the *Nb-1*-expressing cells in the pupal worker brain. Triple labeling by in situ hybridization of *Nb-1* RNA, BrdU immunohistochemistry, and nuclear staining with DAPI was performed using serial coronal sections of the brain of a single worker pupa at the P2 stage injected with BrdU. (**A**) Triple labeling using an antisense riboprobe for *Nb-1* RNA (magenta), anti-BrdU antibody (green), and DAPI (blue). The simultaneous differential interference contrast (DIC) image is also shown (grey). White arrowheads show the areas for *Nb-1*-expressing MB cells. (**B**) Triple labeling using a sense riboprobe for *Nb-1* RNA (magenta), normal IgG (green), and DAPI (blue) as a negative control. (**C–G**) Magnified views of the square surrounded with white dotted lines, corresponding to the right MB, in panel (**A**). (**C**) Schematic drawing of the right pupal MB. Each MB contains two calyces connected to an α-lobe (not seen in this panel) and a β-lobe via a pedunculus. The outline of the MB, areas for proliferating MB cells located in the MB calyces, and pedunculus are shown with dotted yellow lines, magenta, and blue, respectively. (**D**) The merged image of in situ hybridization of *Nb-1* RNA [magenta, panel (**E**)], BrdU immunohistochemistry [green, panel (**F**)], and DAPI staining [blue, panel (**G**)]. MB, mushroom body; mCa, medial calyx; lCa, lateral calyx; β, β-lobe; OL, optic lobe; AL, antennal lobe; Re, retina. Scale bars indicate 200 μm (**A, B**) or 100 μm (**D**).

Using an antisense riboprobe against *Nb-1* RNA and an anti-BrdU antibody, we detected specific signals for *Nb-1* RNA and BrdU in the pupal brain at the P2 stage (Fig. 2A). *Nb-1* RNA signals and BrdU immunostaining were absent in the control sections treated with the sense riboprobe and normal IgG (Fig. 2B). BrdU immunostaining was detected in the center of the MB calyces, which is consistent with the results of a previous report^54^. Strong *Nb-1* RNA signals were restricted to cells that were located in the center of the calyces and had BrdU-positive nuclei (Fig. 2C–G), indicating that *Nb-1* was strongly expressed in proliferating cells located in developing MBs.

Proliferating cell clusters are also located in the other regions in the pupal honey bee brains^53,56^. At the prepupal stage, many BrdU-positive nuclei were observed in the developing OLs (Fig. S12A–C). As described above, two *Nb-1*-expressing cell clusters were detected: one cluster with strong expression was located in the dorsal and ventral parts of the outer layer, and the other was situated in the middle part of the outer layer (Fig. S3H). In these *Nb-1*-expressing cells, BrdU-positive nuclei were observed (Fig. S12B, C). Moreover, a cell cluster that expressed *Nb-1* and was labeled with BrdU was detected in the MBs at the PP stage (Fig. S12E). *Nb-1* expression was also detected in a BrdU-positive cell cluster located posteriorly above the protocerebral neuropil (Fig. S12F). At the P1 stage, nuclei labeled with BrdU were present in the cell clusters expressing *Nb-1* located laterally in the ALs (Fig. S13D). Many BrdU-positive cells, however, did not express *Nb-1*. These results indicated that *Nb-1* is expressed in a subpopulation of proliferating cells in the developing pupal brain. In addition, although BrdU immunolabeling was detected in other *Nb-1*-expressing cell clusters surrounding the neuropil of the protocerebrum (Fig. S13C, D; white arrowheads), *Nb-1*-expressing cells lacking BrdU-positive nuclei were also detected (Fig. S13C, cyan arrowheads). Thus, not all *Nb-1*-expressing cells in the developing brain proliferate.

### Subcellular localization of *Nb-1* RNA in the proliferating cells

**Figure 3:**
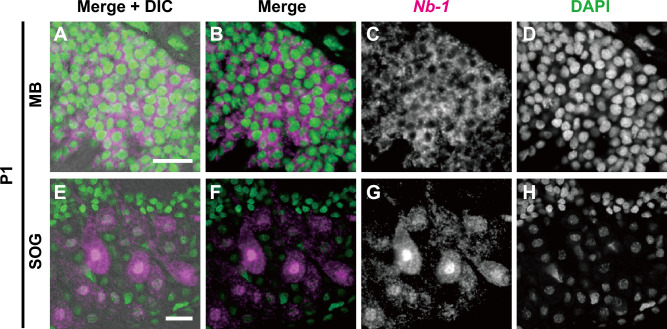
Subcellular localization of *Nb-1* RNA in the pupal worker brain. Double labeling by in situ hybridization using antisense riboprobe for *Nb-1* RNA and nuclear staining with DAPI was performed using brain sections of a single worker pupa at the P1 stage. All the images are single optical sections obtained using a laser-scanning confocal microscope. (**A-D**) High-magnification images of proliferating cells located in the center of developing MB calyces. (**E–H**) High-magnification images of cells that are located in the SOG and correspond to C8 of the adult brain^35^. (**A**, **E**) The merged images of simultaneous DIC images (grey) and panels (**B**, **F**), respectively. (**B**, **F**) The merged image of in situ hybridization of *Nb-1* RNA (magenta) and DAPI staining (green). (**C**, **D** and **G**, **H**) Single-color images for *Nb-1* staining (**C**, **G**) and DAPI staining (**D**, **H**), respectively. MB, mushroom body; SOG, subesophageal ganglion. Scale bars indicate 20 µm.

We previously reported that the subcellular localization of *Nb-1* RNA varies among cells, even within the same cluster, implying that the subcellular localization of *Nb-1* may vary in response to certain neural inputs^35^. Herein, we examined the subcellular localization of *Nb-1* RNA in the proliferating and non-proliferating cells using double staining with fluorescence in situ hybridization and nuclear staining with 4′-6-diamidino-2-phenylindole (DAPI) and confocal laser scanning microscopy. The calyces of the developing MBs contained *Nb-1*-expressing proliferating cells. In these cells, *Nb-1* RNA signals were detected mainly in the cytoplasm but not in the nucleus (Fig. 3A–D). We also observed *Nb-1*-expressing non-proliferating cells at the ventral midline of the SOG (Fig. S13). In these cells, strong *Nb-1* RNA signals were detected in the nuclei (Fig. 3E–H). This finding suggested that *Nb-1* plays a role in the cytoplasm of proliferating cells.

### *Nb-1* expression during oogenesis and early developmental stages

**Figure 4:**
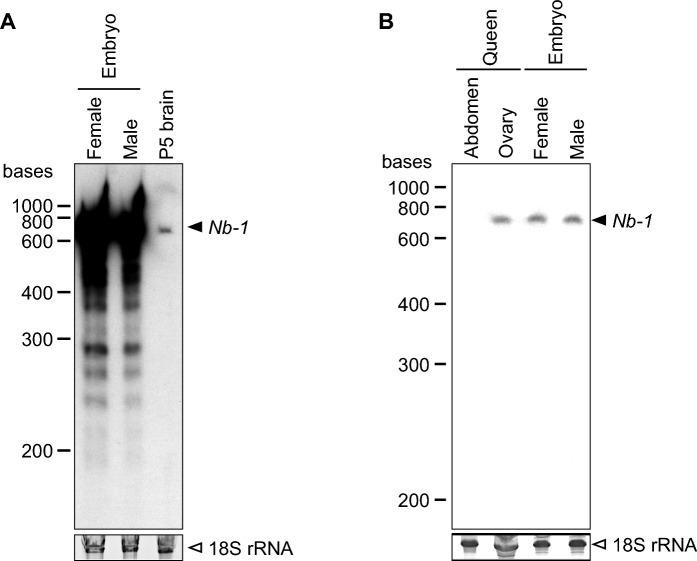
*Nb-1* expression in the female and male embryos, and queen ovary. Northern blotting was performed using total RNAs extracted from the whole female (worker) and male (drone) embryos (each more than 10 individuals) and the whole brains of 4 pupae at the P5 stage (**A**, upper panel), and the ovary and abdomen without ovary, derived from a single queen, and the whole female and male embryos (**B**, upper panel), and antisense riboprobes for *Nb-1* RNA. The same whole female and male embryo samples were used in both panels (A) and (B). The bands for 18S rRNA bands were also detected by staining with ethidium bromide as loading controls (**A, B**, lower panels). The bands corresponding to *Nb-1* RNA and 18S rRNA are shown with black and white arrowheads, respectively, on the right.

Our finding that *Nb-1* expression was detected in the brains of the fifth instar larvae (Fig. 1) suggests that *Nb-1* is already expressed during the earlier developmental stages in which cell proliferation is more active. Wang et al. (2021) recently identified *Nb-1* as the most abundantly expressed lncRNA in female and male honey bee embryos^40^. To explore this further, we first performed section in situ hybridization for *Nb-1* RNA in first-instar larvae and detected *Nb-1* expression almost uniformly in all cells in the sections containing various tissues, suggesting that *Nb-1* is expressed in all cells in the whole body (Fig. S14). We next performed Northern blotting using the total RNA extracted from female and male embryos. Bands for *Nb-1* RNA were detected in both female and male embryos, and their band intensities were much stronger (approximately 50 times) than those obtained from pupal P5 brains (Figs. 4A, S15). Lower weight bands were also observed in both female and male embryos and were likely derived from minor isoforms or degradation products of *Nb-1* RNA. This result indicated that *Nb-1* expression during embryogenesis is much stronger than that during the pupal stages.

**Figure 5:**
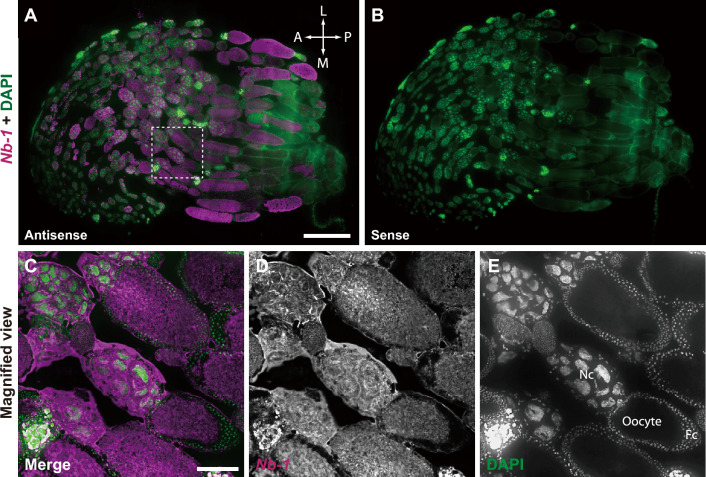
Distribution of the *Nb-1*-expressing cells in the queen ovary. Double labeling by in situ hybridization for *Nb-1* RNA and nuclear staining with DAPI was performed using ovary serial sections derived from a single queen. (**A**) Double labeling using an antisense riboprobe for *Nb-1* RNA (magenta) and DAPI (green). (**B**) Double labeling using a sense riboprobe for *Nb-1* RNA (magenta) and DAPI (green) as a negative control. (**C**–**E**) Magnified views of the square surrounded with white dotted lines in panel (**A**). Nc, nurse cells; Fc, follicle cells. Scale bars indicate 1,000 μm (**A**) and 200 μm (**C**), respectively.

Next, to examine whether *Nb-1* RNA is expressed as a maternal RNA in oocytes, we performed Northern blotting using total RNA extracted from queen ovaries. Strong *Nb-1* expression was detected in the queen ovaries and embryos, although not in other queen abdominal tissues (Figs. 4B, S15). We then analyzed the localization of the *Nb-1* RNA in ovarian cells using in situ hybridization. Each egg chamber of the ovary contained an internal germ cell cluster comprising a single oocyte and 15 nurse cells. The cluster was surrounded by the epithelium of follicle cells of somatic cell origin^58^. *Nb-1* RNA signals were strongly detected in nurse cells and oocytes, but not in follicle cells (Fig. 5). This finding suggested that *Nb-1* RNA accumulates in oocytes during oogenesis as a maternal RNA and functions immediately after hatching.

### Drone-selective *Nb-1* expression in the retinal photoreceptor cells.

Since strong *Nb-1* expression was detected in embryos of both sexes (Fig. 4B), we next performed Northern blotting analysis using total RNA extracted from the brains of larvae, pupae, and adults of drone honey bees, to examine whether *Nb-1* expression in the brain continues at later developmental stages, even in drones. A single band of *Nb-1* RNA, approximately 700 base long (the same size detected in the worker brain), was detected throughout the drone developmental stages (Figs. S16, S17). No prominent differences in the band intensities were detected at any stage between the drones and workers. This finding suggests that identical *Nb-1* RNA functions not only in the worker brain but also in the drone brain.

**Figure 6:**
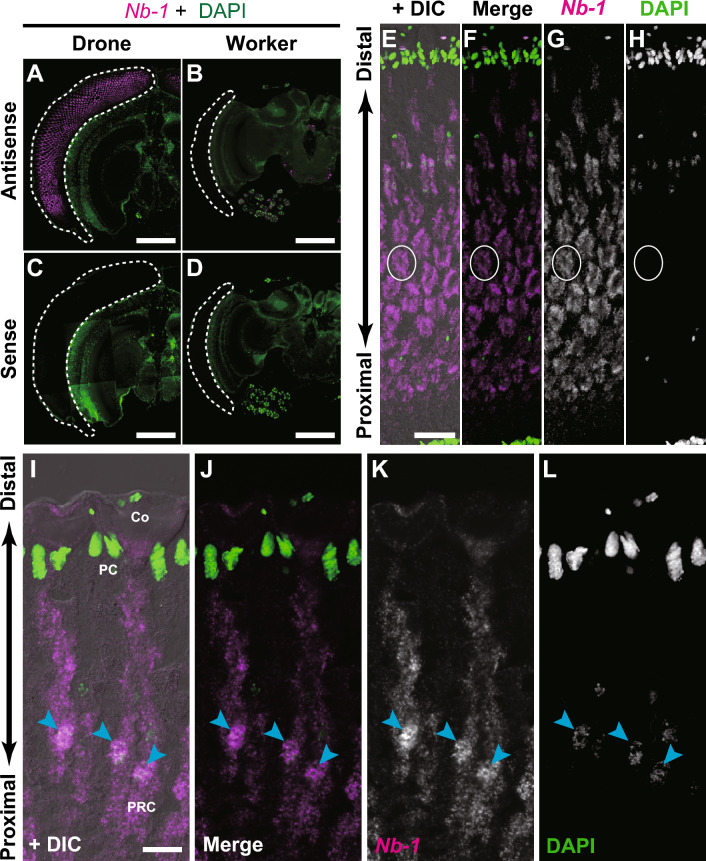
Drone-specific *Nb-1* expression in the retina of adult honey bees. Double labeling by in situ hybridization for *Nb-1* RNA and nuclear staining with DAPI was performed using serial sections of the brain of a single newly emerged drone and those of a single worker. (**A**, **B**) Double labeling using brain sections of a drone (**A**) and a worker (**B**), respectively, and an antisense riboprobe for *Nb-1* (magenta) and DAPI (green). White dotted lines indicate the retinae of compound eyes. (**C**, **D**) Double labeling using brain sections of drones (**C**) and workers (**D**), and a sense riboprobe for *Nb-1* (magenta) and DAPI (green) as a negative control. (**E**–**H**, **I**–**L**) Magnified views of a cross section (**E**–**H**) and a longitudinal section (**I**–**L**) of the drone retina, respectively. (**E**, **I**) The merged image of simultaneous DIC (grey) and panels (**F**, **J**), respectively. (**F**, **J**) The merged image of in situ hybridization of *Nb-1* RNA (magenta) and DAPI staining (green), respectively. (**G**, **H**, **K**, **L**). Single-color images of *Nb-1* RNA and DAPI are presented in panels (**G**, **K**) and (**H**, **L**), respectively. White circles in panels (**E**–**H**) indicate cross views of single ommatidia, respectively. Co, cone cell; PgC, pigment cell; PRC, photoreceptor cell; Rh, rhabdom. Scale bars indicate 500 μm (**A**–**D**), 50 μm (**E**) and 20 μm (**I**).

To compare the distribution patterns of *Nb-1*-expressing cells in the brains of adult workers and drones, we performed in situ hybridization analysis. Considering that the *Nb-1* expression level in the worker brains decreases in an age-dependent manner^35^, we used newly emerged drones for the analysis to avoid any effects of aging. Unexpectedly, strong *Nb-1* expression was detected in the drone retina of compound eyes (Fig. 6A–D). Drone compound eyes are compartmentalized into a male-specific dorsal part and ventral regions resembling the worker eye with different spectral sensitivities^15^. Drone-specific *Nb-1* expression was detected in both the dorsal and ventral areas of the drone retina (Fig. 6A–D), suggesting that *Nb-1* is not related to sex-specific sensitive compartmentalization in the compound eye.

The honey bee compound eye comprises three types of ommatidia, each of which contains nine photoreceptor cells: in addition to the common six green receptor cells, type I ommatidia contain one ultraviolet and one blue receptor cells, type II ommatidia contain two ultraviolet receptor cells, and type III ommatidia contain two blue receptor cells^59,60^. To determine which photoreceptor cells expressed *Nb-1*, we examined the *Nb-1* expression using confocal scanning laser microscopy. Because the honey bee compound eye has a curved structure, we could capture both the cross and longitudinal views of the ommatidia from the coronal sections of the drone compound eyes. The cross-sectional view revealed many annular staining patterns (Fig. 6E–H), indicating that *Nb-1* was expressed in all types of photoreceptor cells in each ommatidium. To examine the subcellular localization of *Nb-1* RNA in the photoreceptor cells of the drone retina, we focused on a longitudinal view of the ommatidia. In all photoreceptor cells, *Nb-1* RNA signals were mainly confined to the nucleus (Fig. 6I–L), suggesting that *Nb-1* RNA functions mainly in the nucleus of the photoreceptor cells of the drone retina.

### *Nb-1* expression in the developing drone pupal brains.

Next, to compare the distribution of *Nb-1*-expressing cells in the developing brains of workers and drones, we performed in situ hybridization analysis using drone pupae with pigmented leg joints, which correspond to the P5 stage of the worker, and an antisense riboprobe for *Nb-1* RNA. The *Nb-1* expression pattern in the drone pupal brain was similar to that in the pupal worker brain, except for a strong expression in the retina and OLs (Fig. S18). In the MBs, cell clusters strongly expressing *Nb-1* were located in the center of the developing calyces, and those weakly expressing *Nb-1* were observed on both sides of the calyces (Fig. S18B, F, K). *Nb-1*-expressing cells were also observed surrounding the neuropils of the ALs and subesophageal ganglion (Fig. S18D, H, P). Moreover, other *Nb-1*-expressing cells were located in the posterior dorsal part of the brain, corresponding to C1 and C2 in the adult worker brain (Fig. S18C, G)^35^. Other *Nb-1*-expressing cells were detected lateral to the protocerebrum (Fig. S18L) and at the posterior region of the OLs (Fig. S18T). Retinal cells of the developing drone brain strongly expressed *Nb-1* (Fig. S18S), whereas the retinal cells of workers at the P5 stage only weakly expressed *Nb-1*, compared with the other brain areas at the P5 stage (Fig. S6). Furthermore, a strong expression of *Nb-1* was detected in the outer layer of the OL (Fig. S18O). Thus, we conclude that *Nb-1* is expressed in a drone-selective manner in the retina of the pupal brain, suggesting that *Nb-1* has a potential drone-specific role in the development and/or function of the visual system.

## Discussion

### *Nb-1* expression in the developing pupal brains suggests its role in neural development and neurogenesis

In the present study, we found that *Nb-1* expression in the worker brain decreased during the pupal and adult stages (Fig. 1). In situ hybridization analysis revealed that *Nb-1* expression was widespread in late larval and early pupal brains, including developing MBs, and was restricted to a small number of cells in the adult brains (Figs. S2–10). In the pupal brain, *Nb-1* RNA was mainly localized to the cytoplasm of proliferating cells, including MB neuroblasts, whereas it was also detected in the nuclei of non-proliferating cells in some regions of the pupal brain (Figs. 3, S13). It is possible that *Nb-1* expressing non-proliferating cells in the pupal brain represent differentiating neural progenitors or postmitotic cells. Therefore, *Nb-1* appears to be expressed in both the developing and differentiating cells in the pupal brain.

*Nb-1* RNA signals were enriched in the cytoplasm of proliferating MB cells, which are localized to the center of the MB calyces (Figs. 2, 3). Considering that elaborate MBs are assumed to be important for the expression of social behaviors and/or advanced brain functions in honey bees^19–26,30,31^, *Nb-1* RNAs may play honey bee-specific roles in the development of elaborate MBs by regulating cell proliferation in the pupal brain. In contrast, *Nb-1* expression in the ALs was retained till the late pupal stages, unlike in MBs (Fig. S10). Although neural cell proliferation in ALs is completed at an earlier stage than in MBs, the formation of olfactory glomeruli continues till the late pupal stages^55,61^. *Nb-1*-expressing cells were restricted to the outer layer of the AL neuropil, which are assumed to be AL projection neurons based on their localization^52,55^. AL projection neurons project neurites to MBs in the adult brain, and neurite extension occurs at later pupal stages^61,62^. *Nb-1* may be expressed in AL projection neurons in the pupal brains and function in the regulation of neurite outgrowth.

### Changes in the subcellular localization of *Nb-1* RNAs might reflect its cell functions

We found that *Nb-1* RNA in proliferating cells tended to be confined to the cytoplasm, whereas those in postmitotic or differentiating cells were localized mainly in the nuclei of the developing larval and pupal brains (Figs. 3 and S13). Therefore, it is possible that the subcellular localization of *Nb-1* RNA changes with the progression of cell differentiation: *Nb-1* RNA may be localized in the cytoplasm during cell proliferation and in the nuclei during neuronal differentiation, such as the neurite outgrowth phase.

Recent studies have shown that lncRNAs modulate transcription through chromatin interactions and remodeling in the nuclei^63,64^, whereas they function to mediate signal transduction and post-transcriptional regulation of gene expression in the cytoplasm^65,66^. Subcellular localization enables distinct functions of the same lncRNA through interactions with distinct protein partners^67^. Therefore, changes in the subcellular localization of *Nb-1* RNA between the nucleus and the cytoplasm may switch its nuclear and cytoplasmic functions. *Nb-1* may be involved in transcriptional regulation in the nuclei of proliferating cells while also being involved in different regulatory processes, such as translational regulation and/or intracellular signaling, when localized in the cytoplasm of non-proliferating cells.

### *Nb-1* is a highly abundant lncRNA during oogenesis and embryogenesis

The present study revealed that *Nb-1* RNA not only accumulated as abundant maternal RNA in the oocyte during oogenesis but was also strongly expressed in the embryos in both sexes (Figs. 4, 5), the latter of which is consistent with a previous report that *Nb-1* RNA accounts for approximately 36.5% of total lncRNA expressed in the honey bee embryos^40^. Considering that Wang et al*.* (2021) reported that *Nb-1* expression increases only in haploid (male) embryos 72 h after egg-laying^40^, these findings suggest that *Nb-1* functions in early embryogenesis. *Nb-1* RNA is likely to be expressed in all cells of the first instar larvae (Fig. S14), which is also consistent with the results of the whole-mount in situ hybridization that showed a robust increase in *Nb-1* expression in drone embryo^40^, suggesting a role for *Nb-1* RNA in the proliferation of various embryonic and larval cells.

Although many maternal lncRNAs have been identified in various animals, their functions remain controversial. For example, the knockdown of the mouse oocyte-specific lncRNA variant Rose RNA causes abnormal oocyte cytokinesis and impaired preimplantation embryo development^68^. In contrast, the knockout of Sirena 1 RNA, which is the most abundant lncRNA in mouse oocytes, does not affect fertility but minorly affects the maternal transcriptome^69^. A recent study reported that most of the lncRNAs, some of which are expressed maternally, are dispensable for zebrafish embryogenesis, viability, and fertility^70^. Therefore, the function of *Nb-1* expressed maternally during oogenesis and embryogenesis needs to be substantiated in future studies.

### Drone-specific retinal expression of *Nb-1* implied its role in the drone visual system

The results revealed drone-specific expression of *Nb-1* RNAs in the adult retina (Figs. 6, S18). Beginning with Xist and roX RNA^1,2^, numerous lncRNAs that are expressed and function in a sex-preferential/specific manner have been identified in various animals^40,71,72^. In addition, many lncRNAs that are expressed preferentially and function in the retina have also been identified in mammals^73,74^. However, to our knowledge, this is the first instance of a lncRNA (*Nb-1)* being expressed in a sex-specific manner in the insect retina.

In situ hybridization revealed strong expression of *Nb-1* in photoreceptor cells of the adult drone retina (Fig. 6). There are two possible explanations for the functions of *Nb-1* RNA in the adult drone retina. One is that *Nb-1* RNA is involved in the physiological regulation of the drone visual system, and the other is that *Nb-1* RNA plays a role in retinal development, even in the early adult stages in drones. Considering that *Nb-1* signals were confined to the nuclei of adult drone photoreceptor cells, similar to those in the non-proliferating cells of the developing worker pupal brains (Fig. S13), the differentiation of the drone retina may not be complete immediately after emergence, as suggested by a previous study^60^, and *Nb-1* RNA may contribute to the maturation of photoreceptor cells. In both cases, *Nb-1* may underlie well-developed drone visual system.

### Possible role of *Nb-1* in expressing multiple developmental, behavioral, and physiological traits associated with the honey bee lifecycle

We previously identified three lncRNAs from the honey bees: Ks-1^32^, AncR-1^33^, and kakusei^34^. Among them, Ks-1 RNA is localized preferentially in the nuclei of small-type Kenyon cells, a certain type of intrinsic MB interneurons, and in the suboesophageal ganglion of the worker honey bee brain, suggesting its neural functions^32^. AncR-1 RNA is localized in the nuclei of the whole brain cortex, sex organs of queens and drones, and hypopharyngeal glands and oenocytes of workers, suggesting diverse organ functions^33^. In contrast, kakusei RNA is a product of an immediate early gene, which is transiently induced in neurons after neural excitation and is localized in the nuclei in the worker brain^34^. Therefore, the function of *Nb-1* RNA appears to differ from those of Ks-1, AncR-1, and kakusei RNAs.

*Nb-1* is only conserved in honey bee species (genus *Apis*) (Table S1), and its expression patterns, revealed in our previous and present studies, seem to be closely associated with the life cycle characteristics of the honey bee. We have previously suggested a regulatory role of *Nb-1* RNA in the age-polyethism of workers mediated by neural modulation^35^. Herein, we suggest that *Nb-1* plays a potential role in regulating the cell proliferation of MB neuroblasts (Figs. 2, S12, S13), contributing to the formation of elaborate MB structures in the honey bee^54^. Moreover, *Nb-1* plays a potential role in embryogenesis as an abundant maternal RNA (Figs. 4, 5)^40^. Finally, *Nb-1* plays a potential role in visual development and/or sensing in the drone retina (Figs. 6 and S18), which underlies a well-developed drone visual system^12,13^. Therefore, *Nb-1* may potentially modulate multiple developmental, behavioral, and physiological traits of honey bees that are closely related to or are even the basis of honey bee sociality.

Although honey bees exhibit highly advanced social behaviors, the honey bee genome has substantially fewer protein-coding genes (approximately 11,000 genes) than those of *D. melanogaster* (approximately 13,500) and *Anopheles gambiae* (approximately 14,000)^14^. Moreover, the basic molecular functions of genes that are believed to be involved in the social behaviors of the honey bee, such as *foraging*^28^, *HR38*^75–78^, and *ecdysone receptor*^25,79–82^, are conserved among insect species. Thus, some unknown genus-specific genes in honey bees may modify the basic regulatory processes, mediated by these protein-coding genes. We speculate that *Nb-1* may potentially play pleiotropic regulatory roles not only in the age-polyethism of workers but also in MB neuroblast proliferation, embryogenesis, and drone visual development and/or sensing, which underlies the honey bee sociality. Further analyses of *Nb-1* RNA functions in the expression of morphogenetic, behavioral, and physiological traits specific to the honey bee using RNAi^83,84^ and/or genome editing techniques^85–87^ will reveal the molecular evolution of lncRNAs that contribute to the acquisition of genus-specific biological traits through genus-specific modification of molecular processes conserved among animal species.

### Supplementary Information

Supplementary Figure 1.

Supplementary Figure 2.

Supplementary Figure 3.

Supplementary Figure 4.

Supplementary Figure 5.

Supplementary Figure 6.

Supplementary Figure 7.

Supplementary Figure 8.

Supplementary Figure 9.

Supplementary Figure 10.

Supplementary Figure 11.

Supplementary Figure 12.

Supplementary Figure 13.

Supplementary Figure 14.

Supplementary Figure 15.

Supplementary Figure 16.

Supplementary Figure 17.

Supplementary Figure 18.

Supplementary Legends.

Supplementary Table 1.

Supplementary Table 2.

## Data Availability

All data generated or analyzed during this study are included in this published article and its supplementary information files.
