# Supplementary figures and images for: Unique spatially and temporary-regulated/sex-specific expression of a long ncRNA, Nb-1, suggesting its pleiotropic functions associated with honey bee lifecycle
Source: Sci Rep. 2024 Apr 15;14:8701. doi: 10.1038/s41598-024-59494-6 (PMC11018616; doi:10.1038/s41598-024-59494-6)

**Fig. S1**

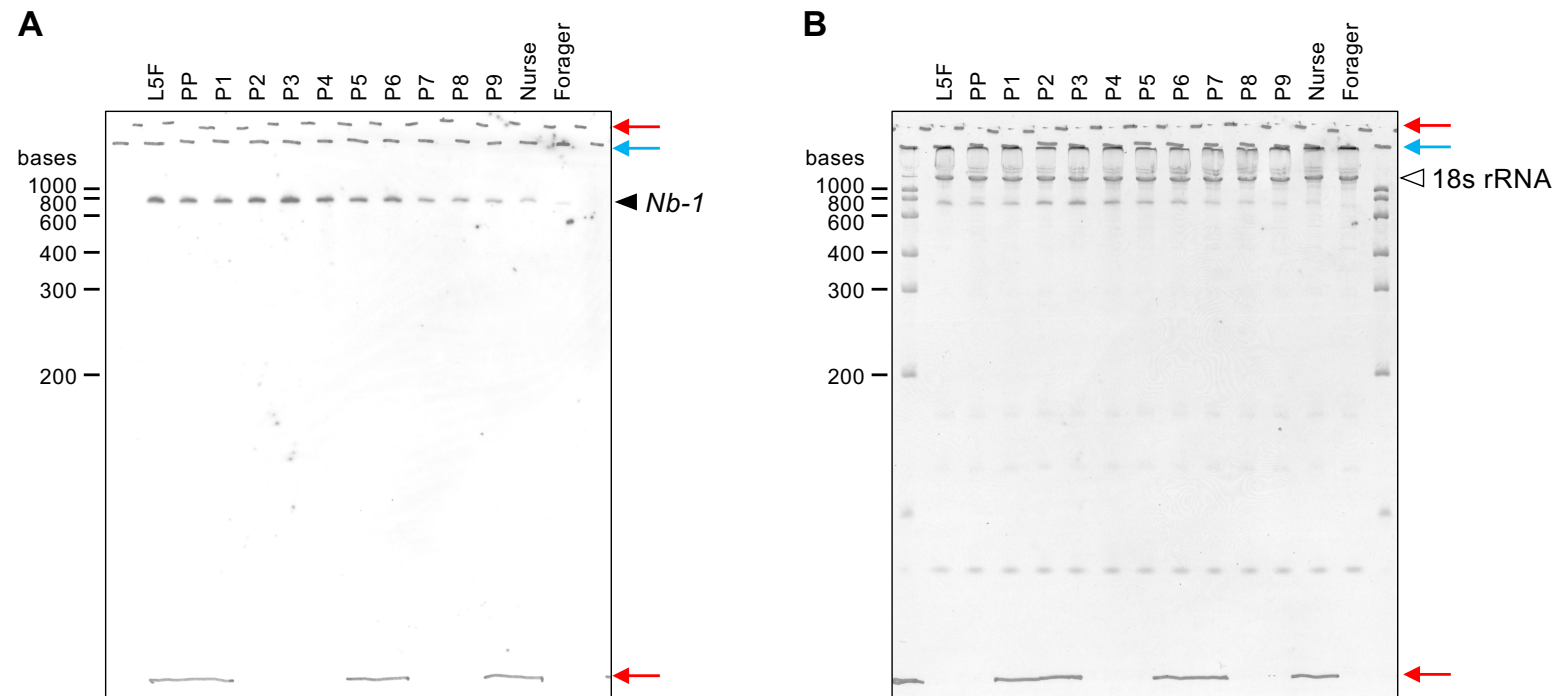

Supplement: Supplementary file 1 — Supplementary Figure 1. [file 41598_2024_59494_MOESM1_ESM.pdf]

**Fig. S2**

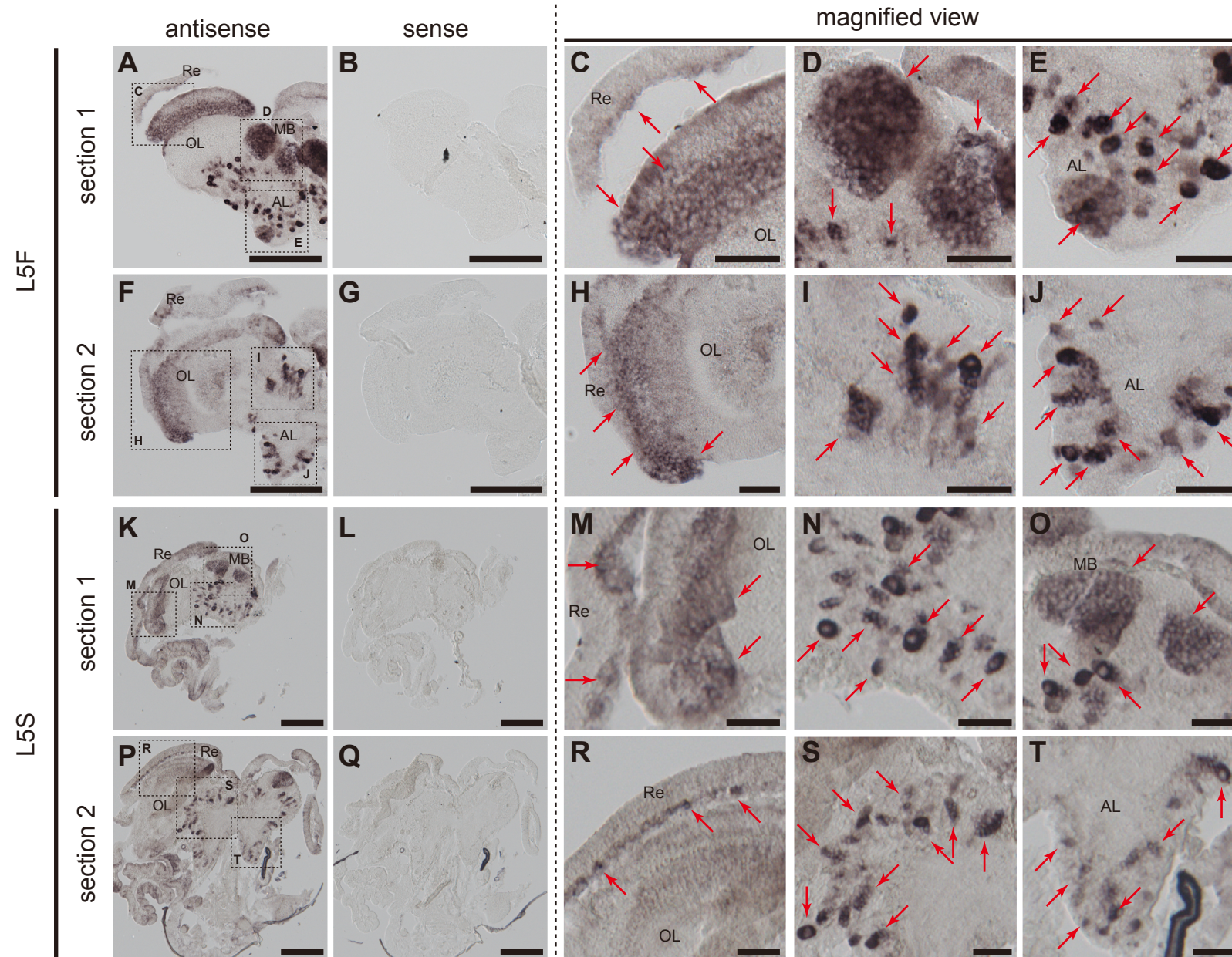

Supplement: Supplementary file 2 — Supplementary Figure 2. [file 41598_2024_59494_MOESM2_ESM.pdf]

**Fig. S3**

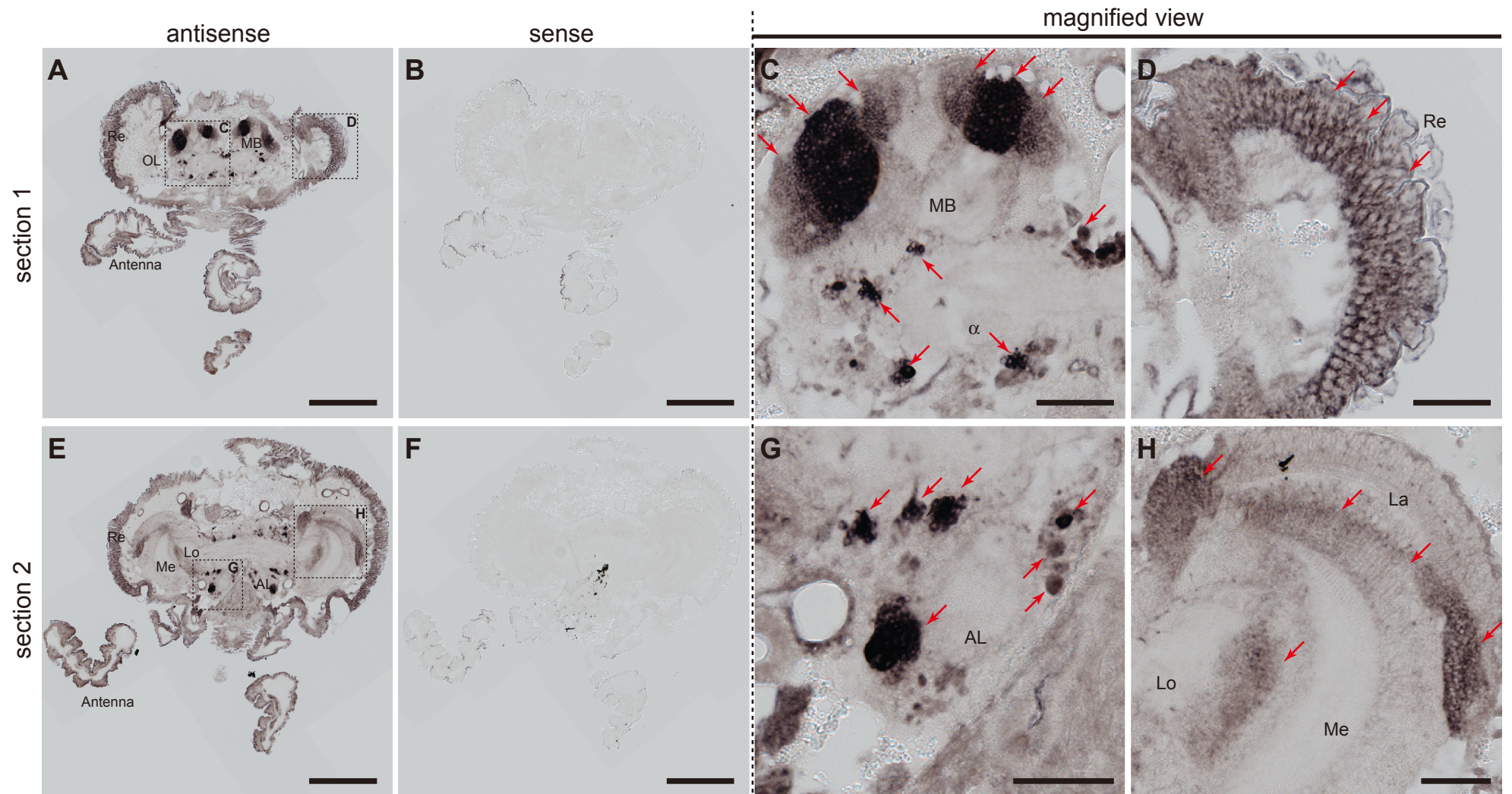

Supplement: Supplementary file 3 — Supplementary Figure 3. [file 41598_2024_59494_MOESM3_ESM.pdf]

**Fig. S4**

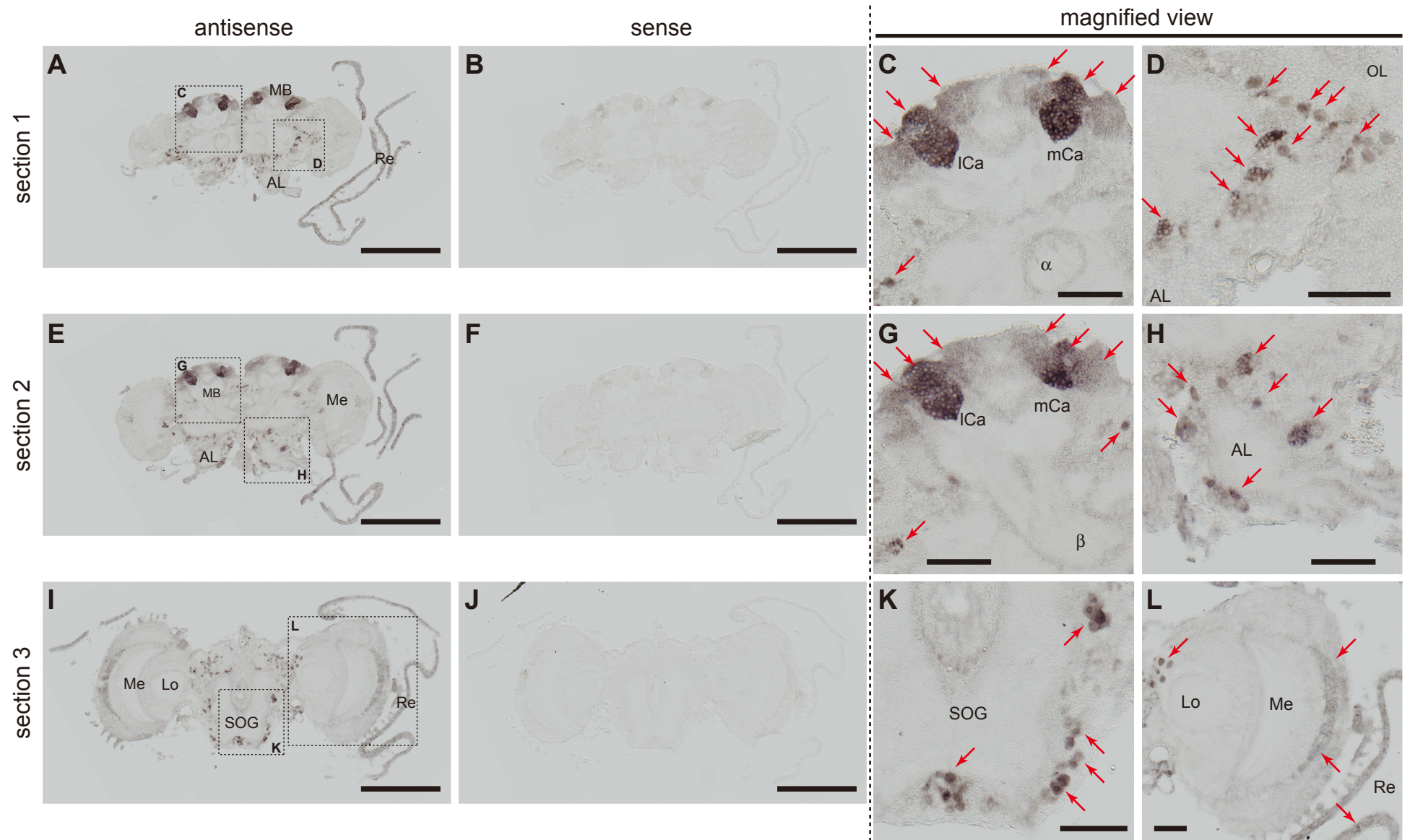

Supplement: Supplementary file 4 — Supplementary Figure 4. [file 41598_2024_59494_MOESM4_ESM.pdf]

**Fig. S5**

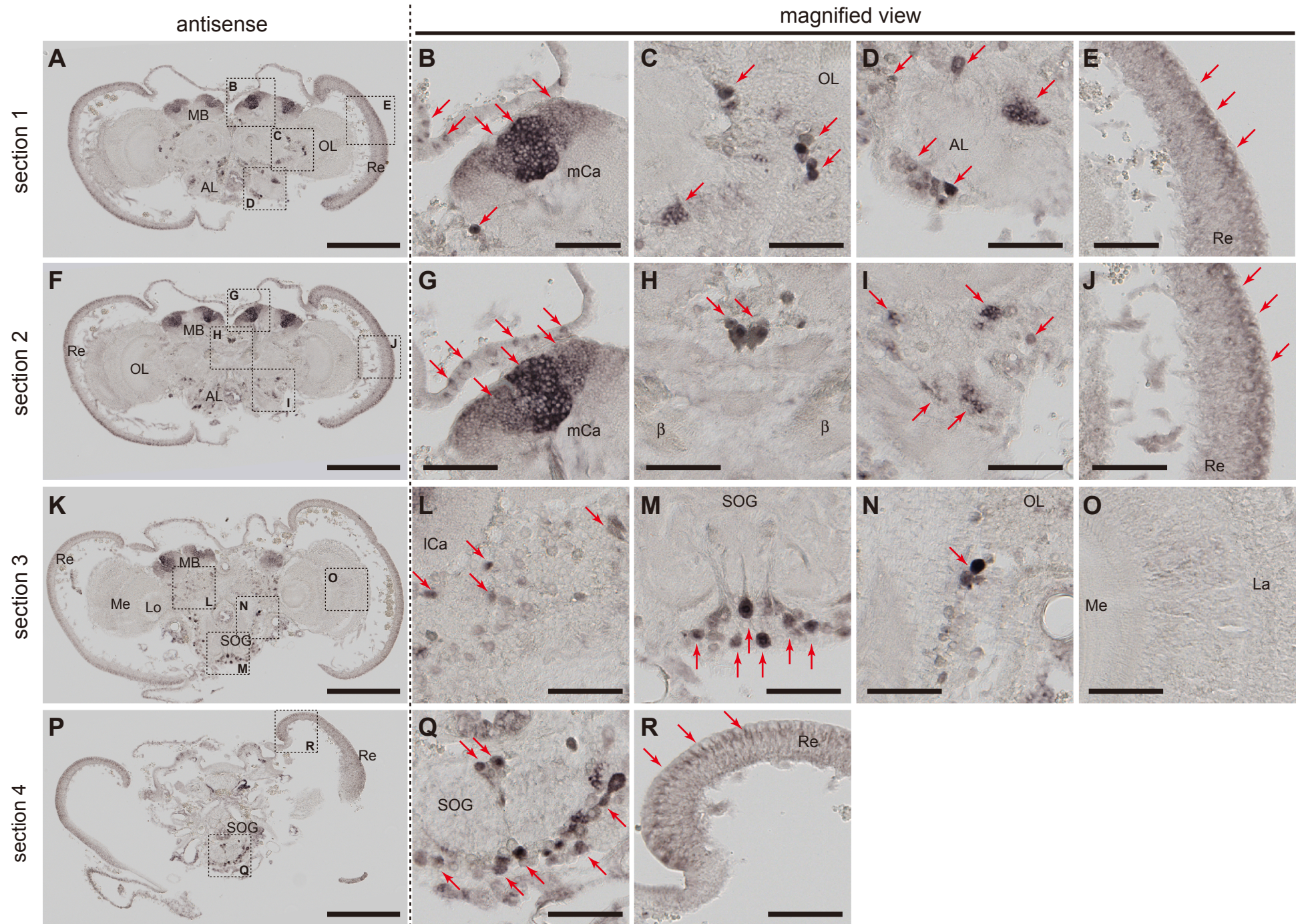

Supplement: Supplementary file 5 — Supplementary Figure 5. [file 41598_2024_59494_MOESM5_ESM.pdf]

**Fig. S6**

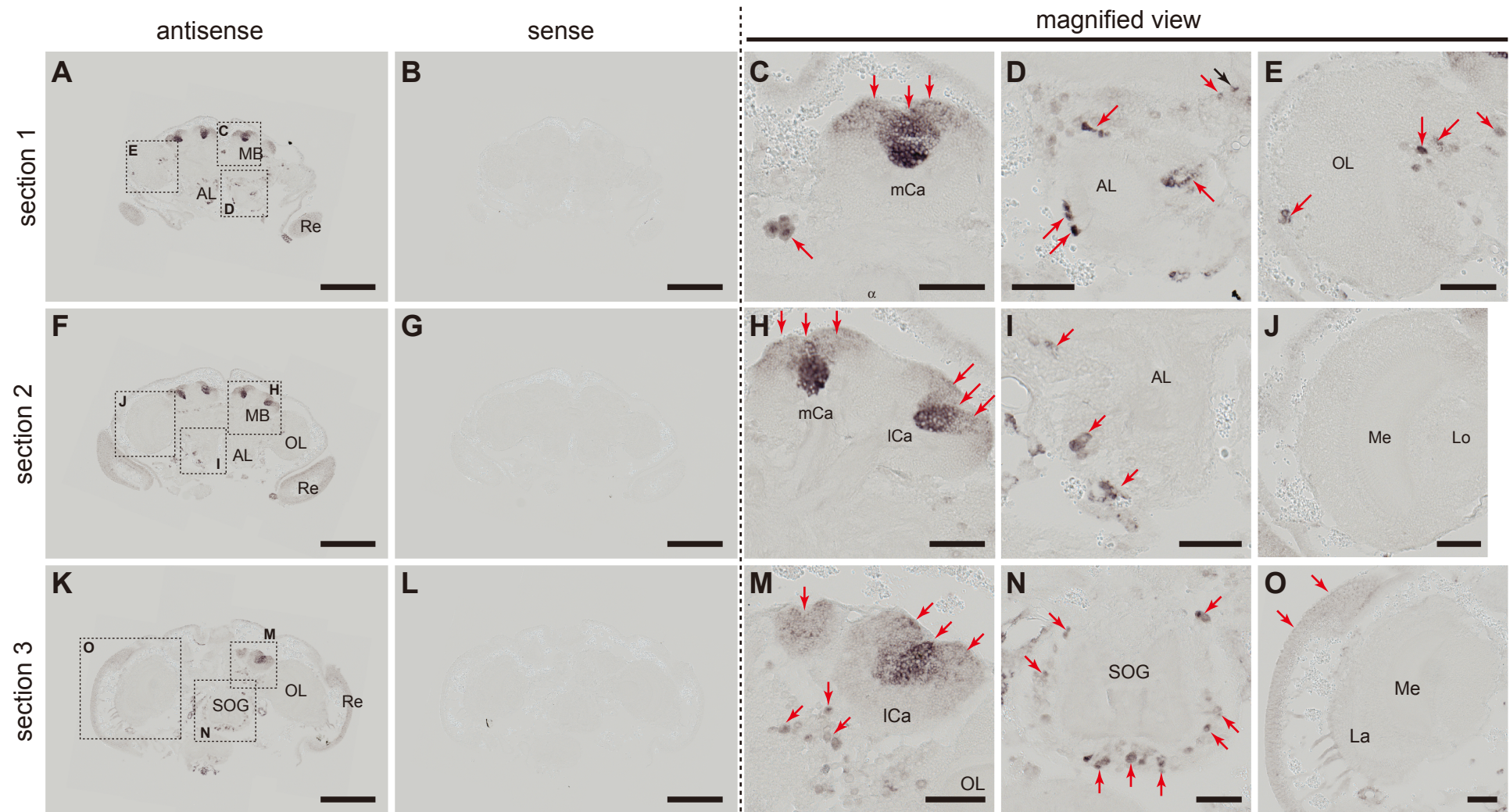

Supplement: Supplementary file 6 — Supplementary Figure 6. [file 41598_2024_59494_MOESM6_ESM.pdf]

**Fig. S7**

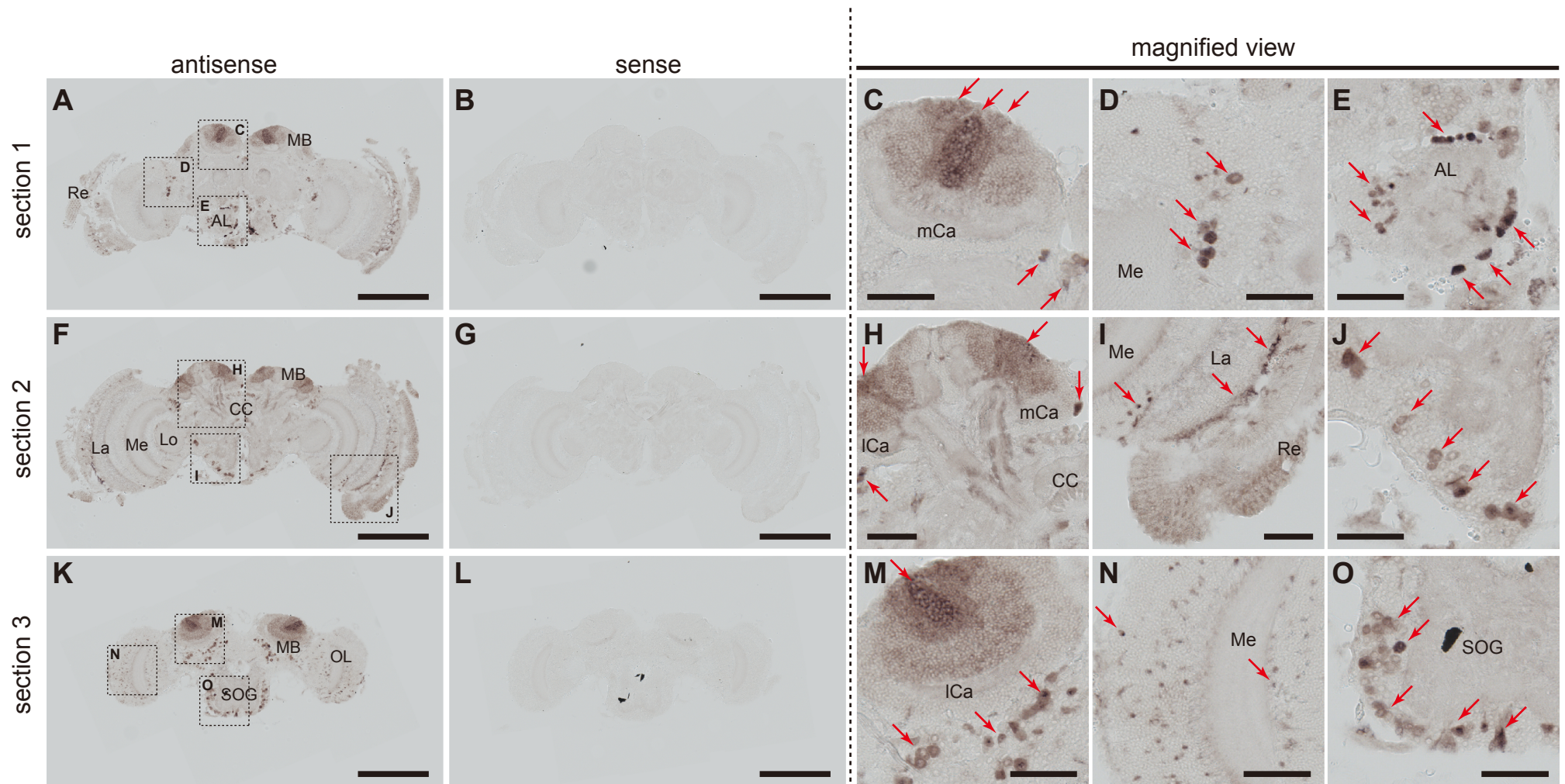

Supplement: Supplementary file 7 — Supplementary Figure 7. [file 41598_2024_59494_MOESM7_ESM.pdf]

**Fig. S8**

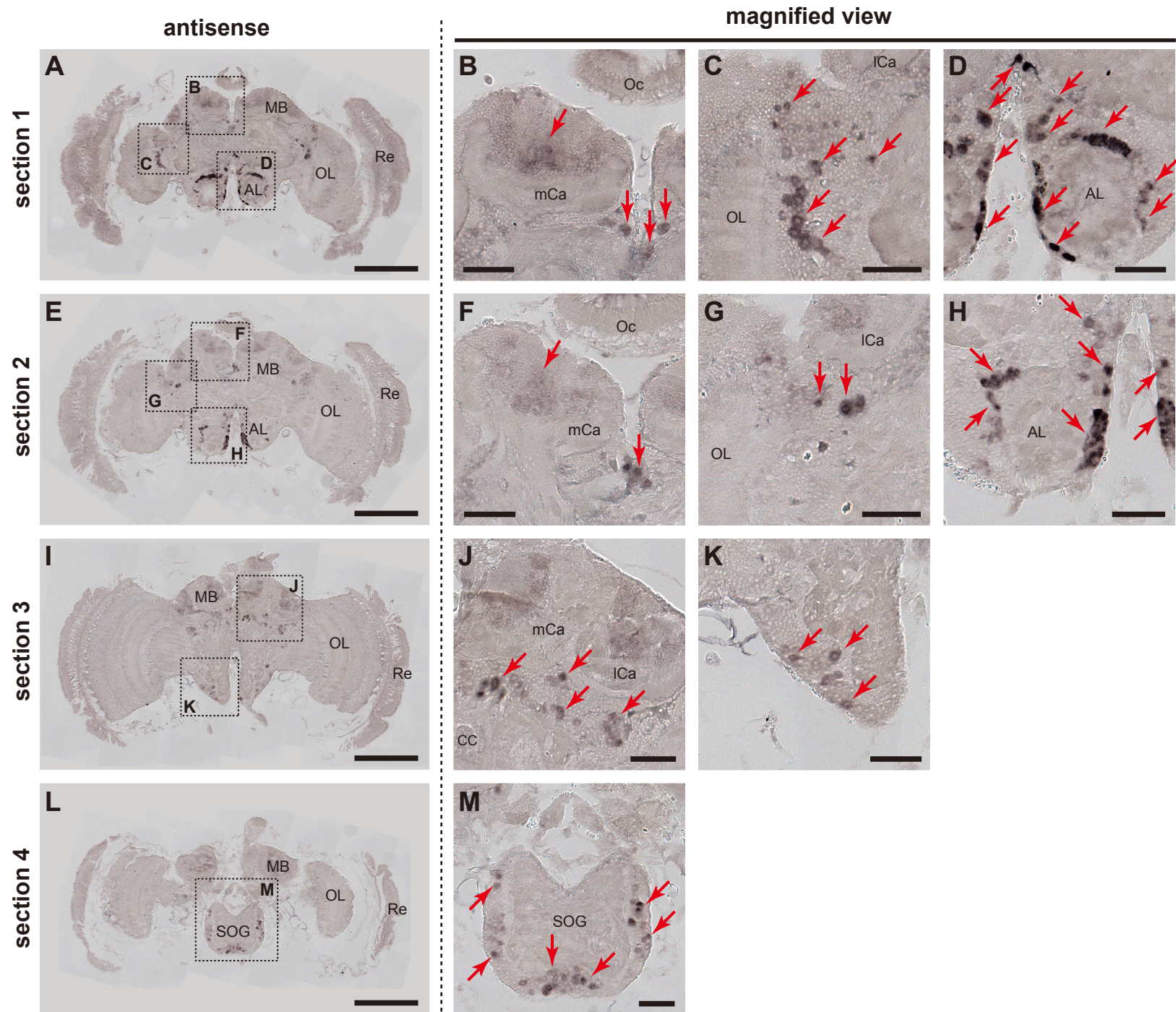

Supplement: Supplementary file 8 — Supplementary Figure 8. [file 41598_2024_59494_MOESM8_ESM.pdf]

**Fig. S9**

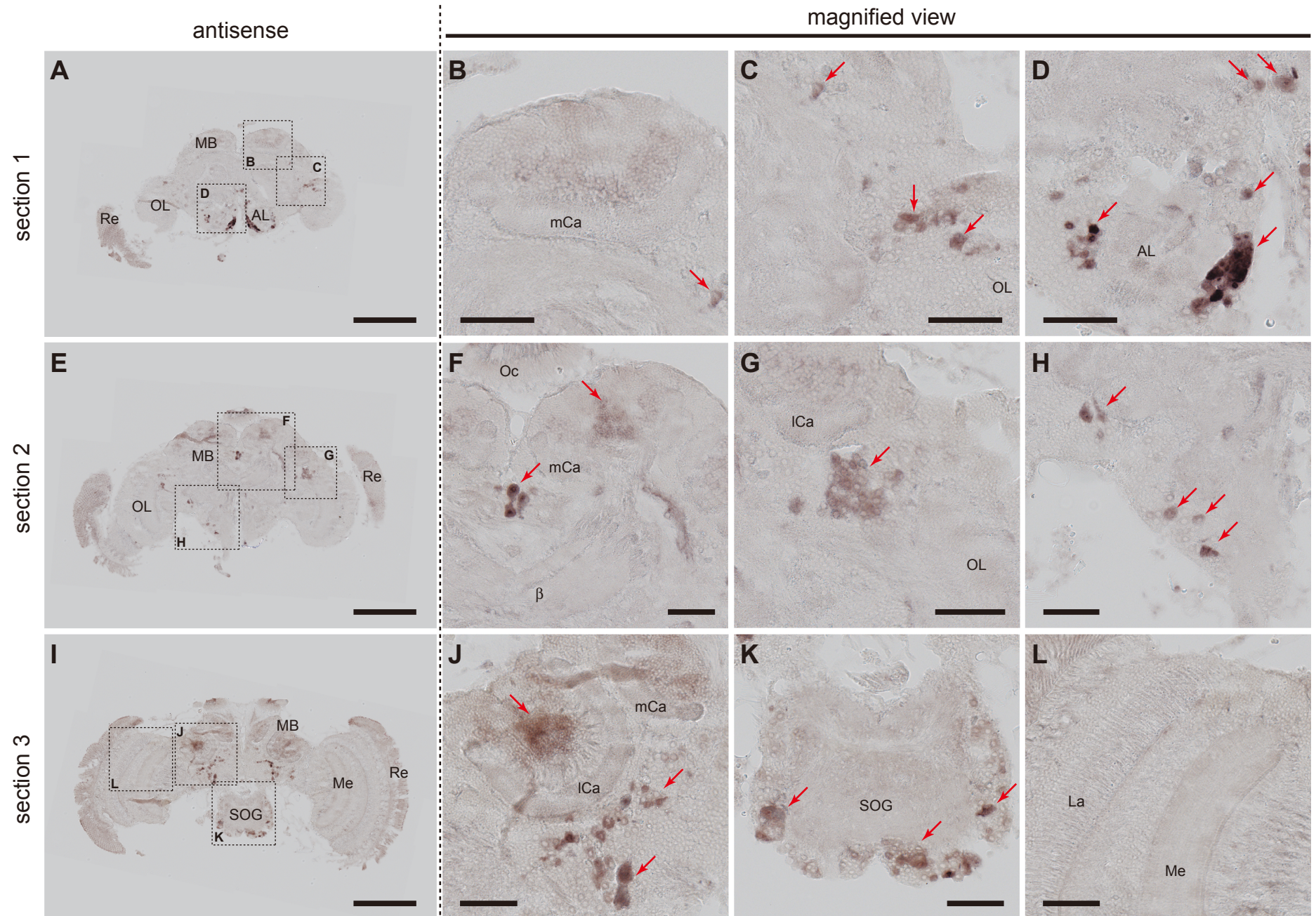

Supplement: Supplementary file 9 — Supplementary Figure 9. [file 41598_2024_59494_MOESM9_ESM.pdf]

Fig. S10

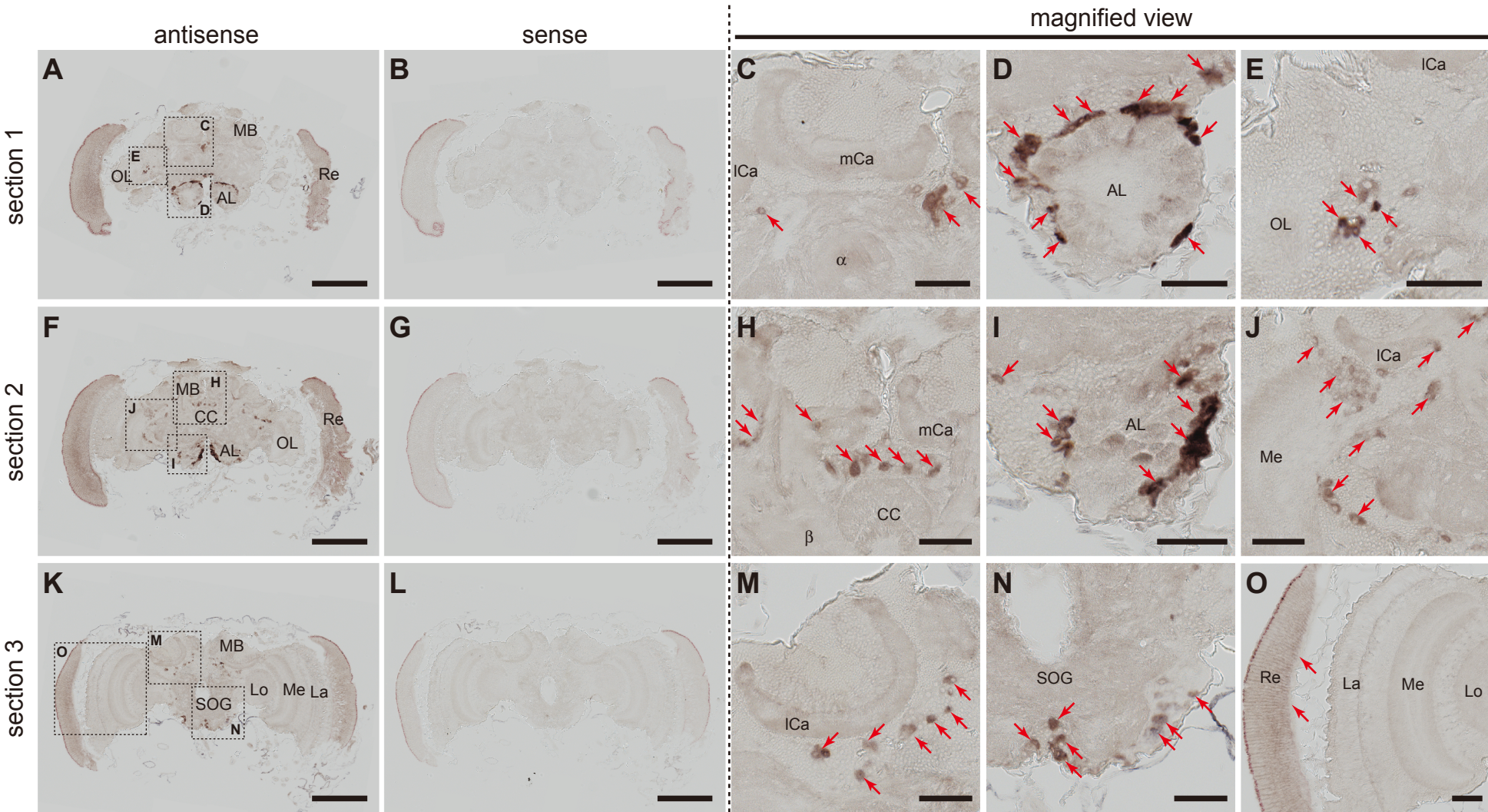

Supplement: Supplementary file 10 — Supplementary Figure 10. [file 41598_2024_59494_MOESM10_ESM.pdf]

Fig. S11

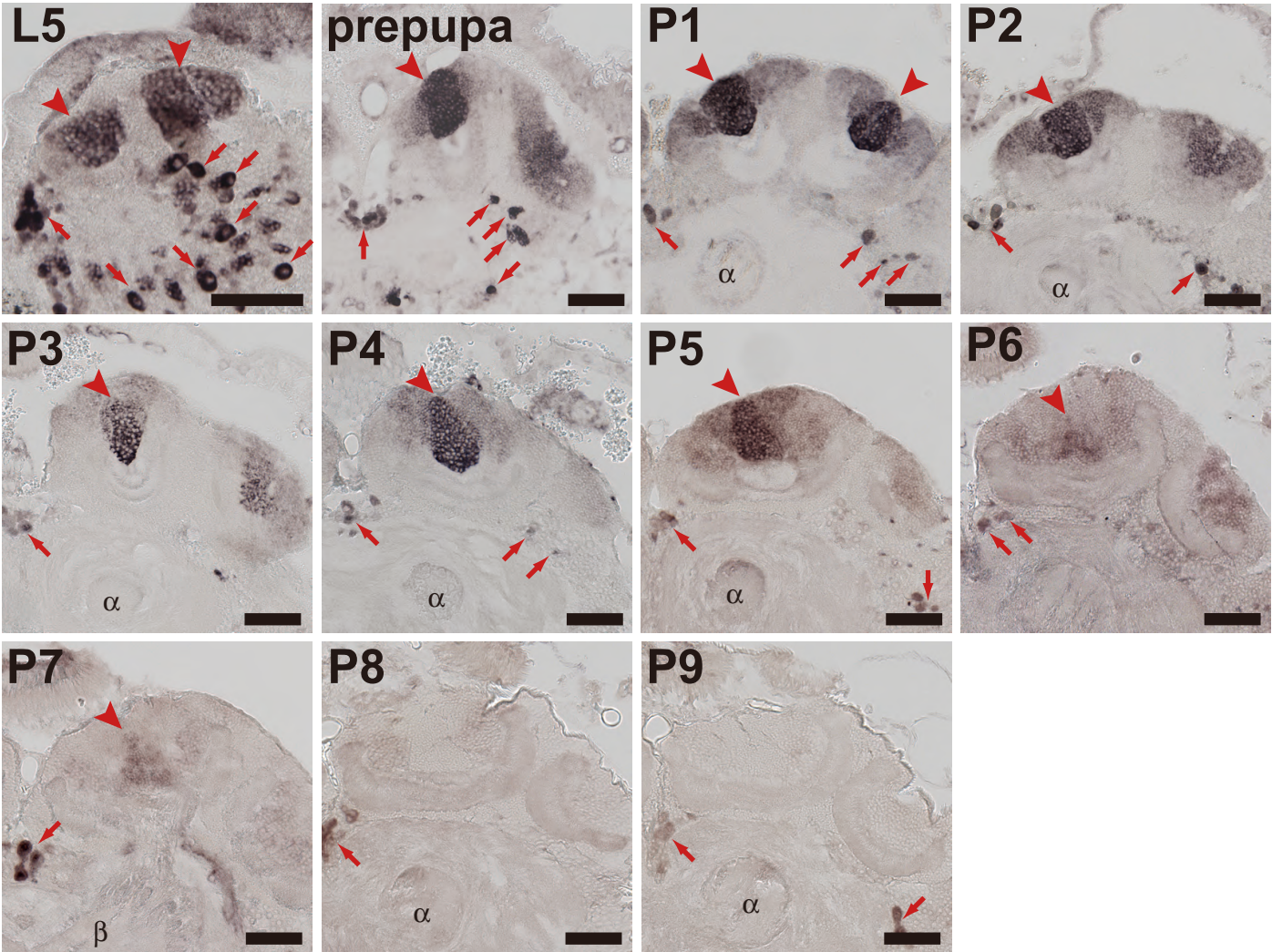

Supplement: Supplementary file 11 — Supplementary Figure 11. [file 41598_2024_59494_MOESM11_ESM.pdf]

**Fig. S12**

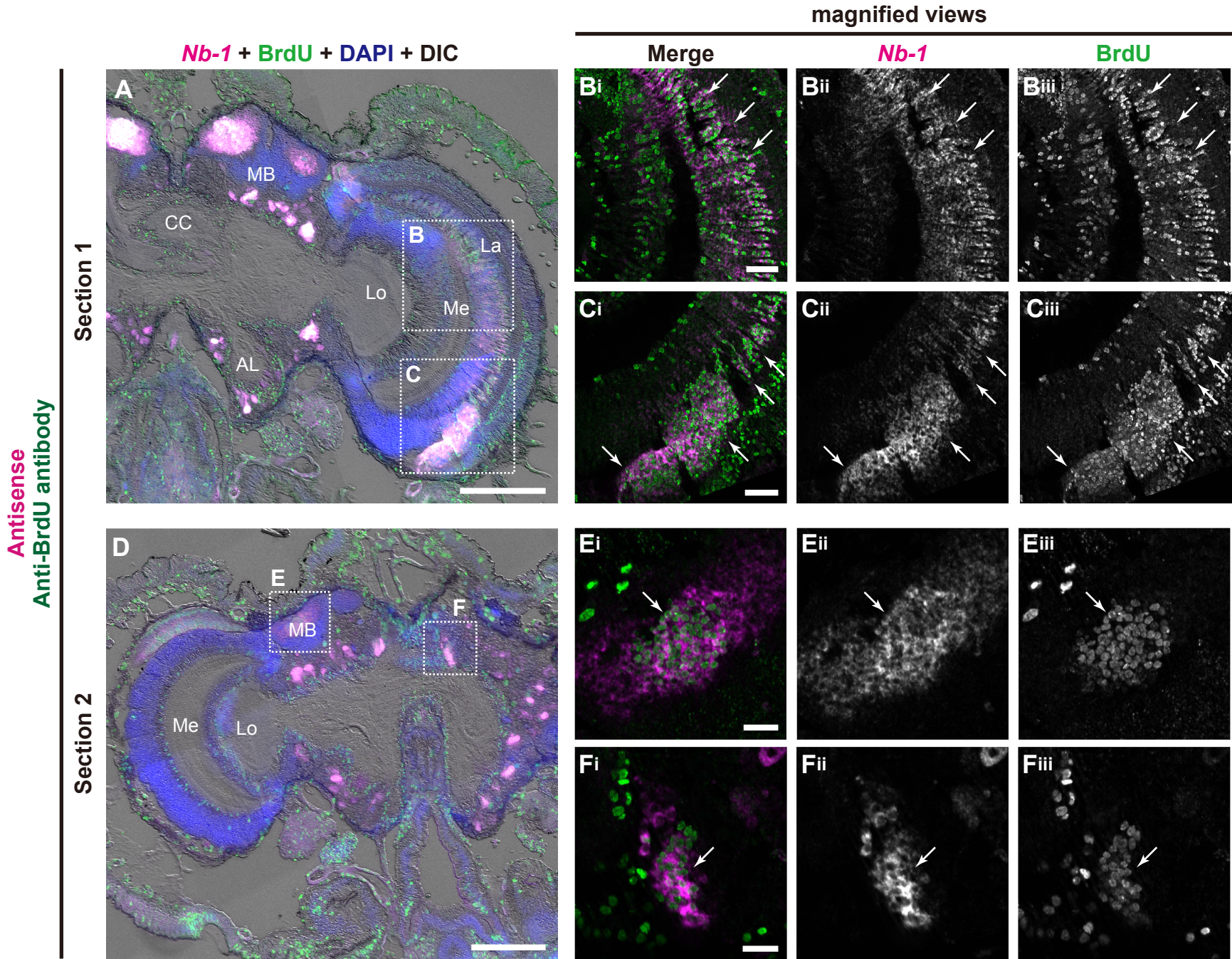

Supplement: Supplementary file 12 — Supplementary Figure 12. [file 41598_2024_59494_MOESM12_ESM.pdf]

Fig. S13

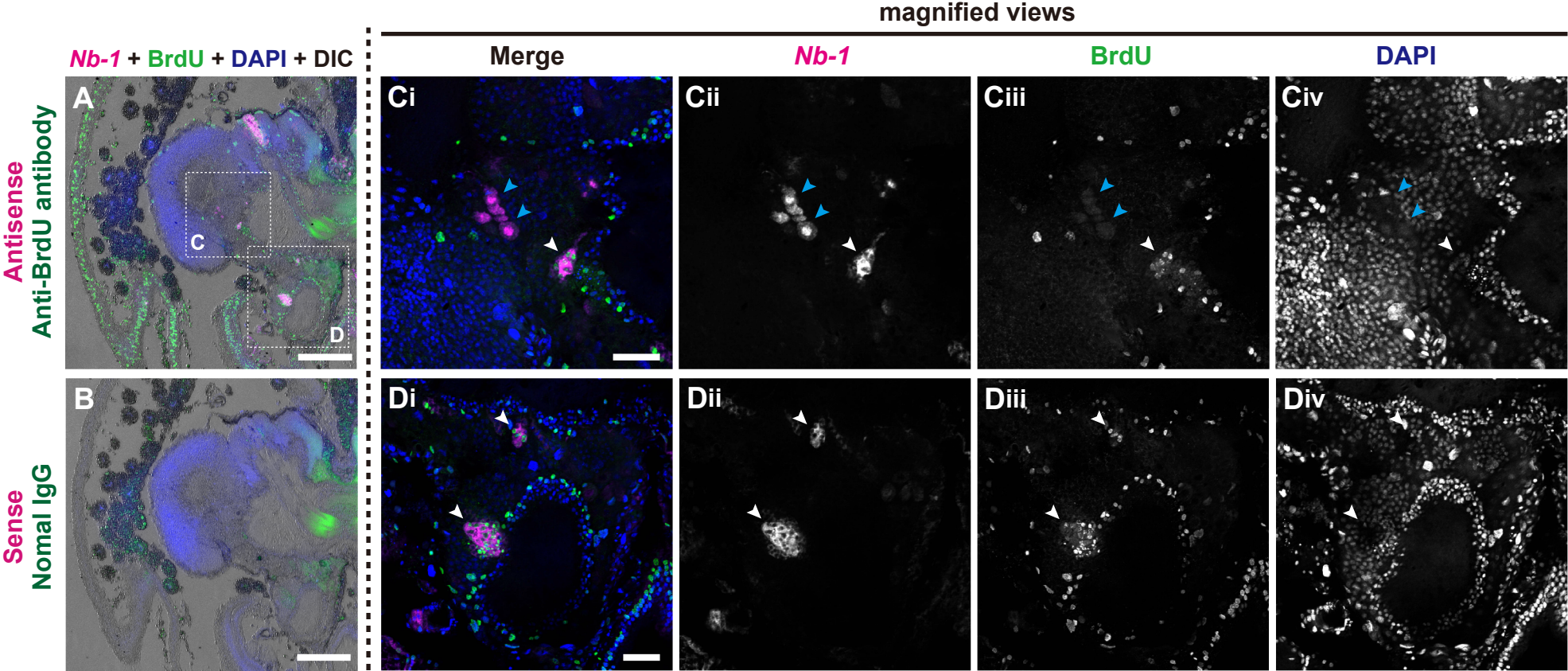

Supplement: Supplementary file 13 — Supplementary Figure 13. [file 41598_2024_59494_MOESM13_ESM.pdf]

**Fig. S14**

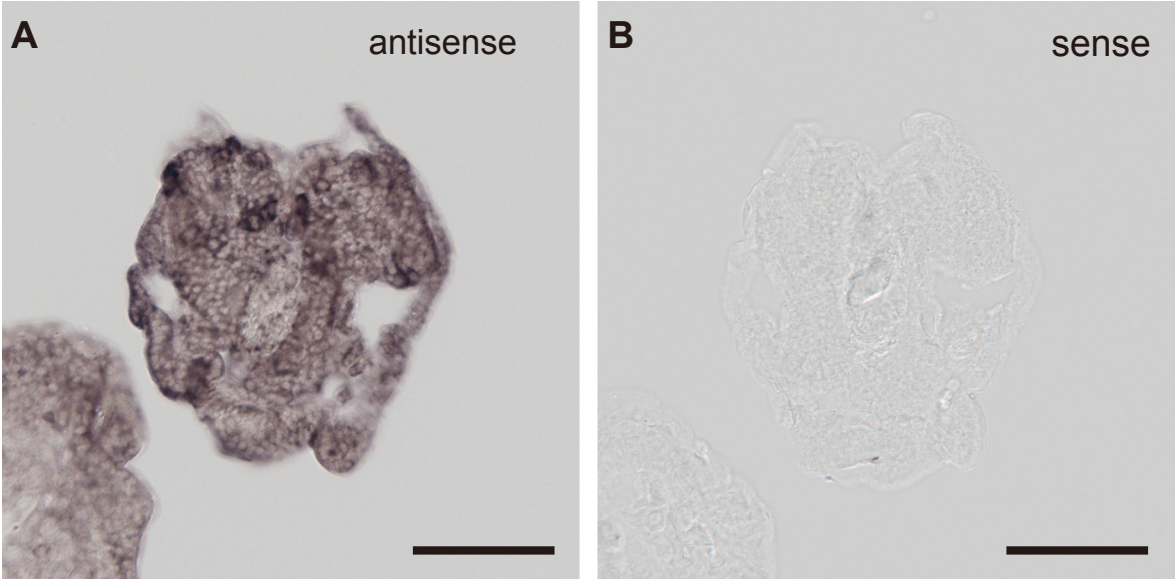

Supplement: Supplementary file 14 — Supplementary Figure 14. [file 41598_2024_59494_MOESM14_ESM.pdf]

Fig. S15

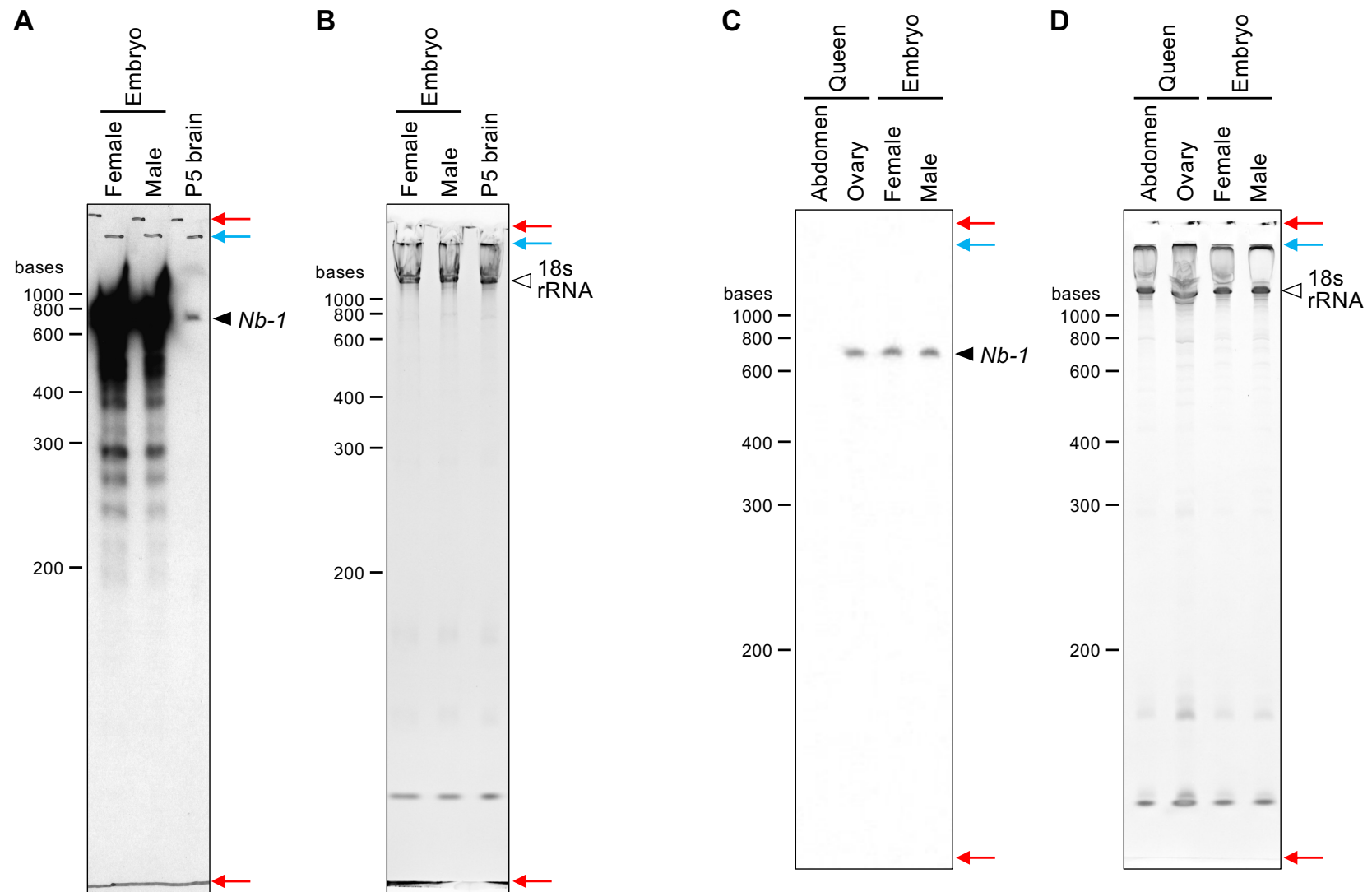

Supplement: Supplementary file 15 — Supplementary Figure 15. [file 41598_2024_59494_MOESM15_ESM.pdf]

**Fig. 16**

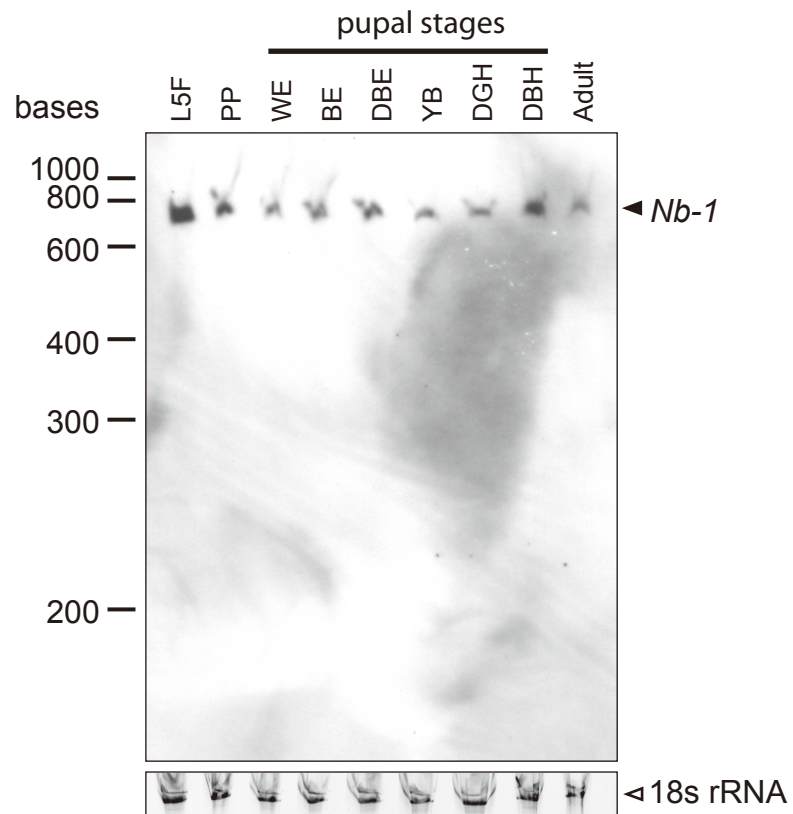

Supplement: Supplementary file 16 — Supplementary Figure 16. [file 41598_2024_59494_MOESM16_ESM.pdf]

**Fig. S17**

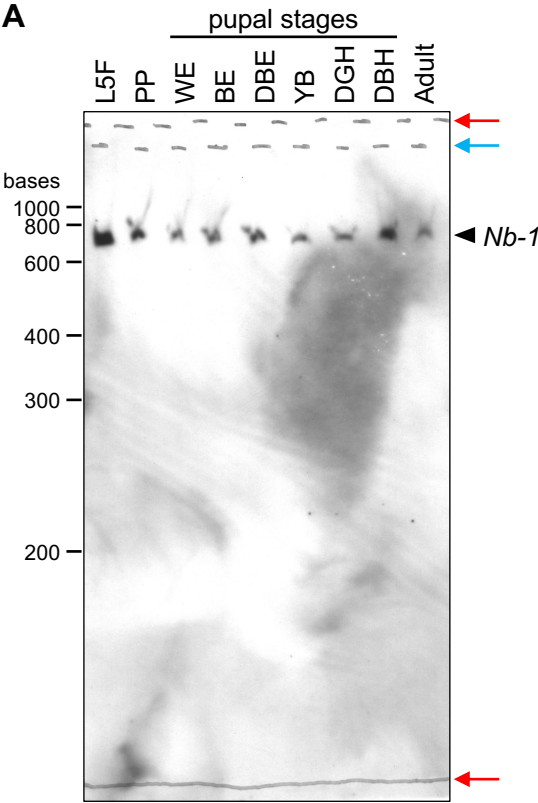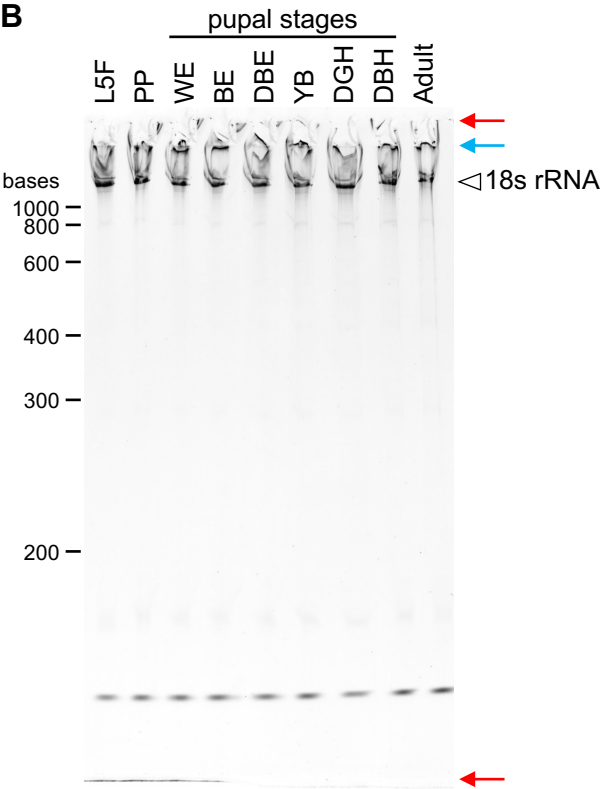

Supplement: Supplementary file 17 — Supplementary Figure 17. [file 41598_2024_59494_MOESM17_ESM.pdf]

**Fig. S18**

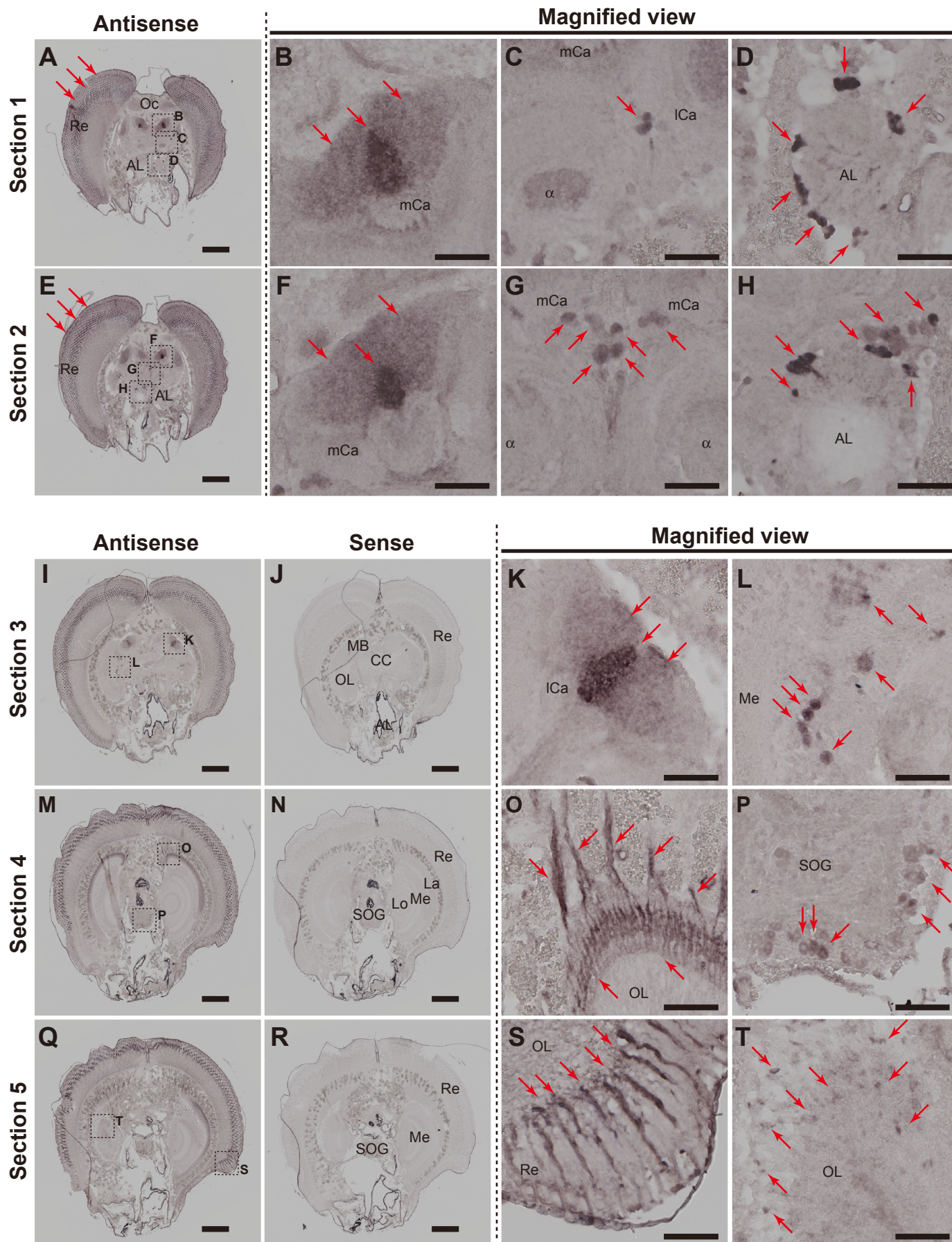

Supplement: Supplementary file 18 — Supplementary Figure 18. [file 41598_2024_59494_MOESM18_ESM.pdf]
