# Supplementary material for: Unique spatially and temporary-regulated/sex-specific expression of a long ncRNA, Nb-1, suggesting its pleiotropic functions associated with honey bee lifecycle
Source: Sci Rep. 2024 Apr 15;14:8701. doi: 10.1038/s41598-024-59494-6 (PMC11018616; doi:10.1038/s41598-024-59494-6)
Supplement: Supplementary file 19 — Supplementary Legends. [file 41598_2024_59494_MOESM19_ESM.docx]

**Legends for Supplementary Figures**

**Figure S1.** **Full-length fluorescence images of the blot and the gel for Fig. 1.**

(A) Full-length fluorescence image of the blot for the upper panel of Fig. 1. Samples are total RNAs extracted from the brains of workers at the 5th instar larval feeding stage (L5F), prepupal stage (PP), pupal stages (P1-P9), and adult stages (nurse and forager). The top and bottom edges of the gel, the sample loaded line, and the bands for *Nb-1* were indicated with red arrows, a blue arrow, and an arrowhead, respectively, on the right. (B) Full-length fluorescence image of the gel stained with ethidium bromide for the lower panel of Fig. 1. The top and bottom edges of the gel, the sample loaded line, and the bands for 18S rRNA were indicated with red arrows, a blue arrow, and an arrowhead, respectively. Note that the right and leftmost lanes indicate molecular weight markers.

**Figure S2. Distribution of *Nb-1*-expressing cells in the brain of five-instar worker larvae.**

*In situ* hybridization was performed using coronal sections of the brain of a single worker larva at the L5F stage (panels **A, B, F, G**) and a single worker larva at the L5S stage (panels K, L, P, and Q) and an antisense (panels **A, F, K, P**) or sense riboprobes (panels **B, G, L, and Q**) for *Nb-1* RNA. Section 1 is anterior to Section 2. (**C-E, H-J, M-O, and R-T**) The magnified views of squares surrounded with dotted lines in panels (**A, F, K, and P**) are shown, respectively. The *Nb-1* RNA signals were indicated by red arrows. MB, mushroom body; OL, optic lobe; AL, antennal lobe; Re, retina. Scale bars indicate 200 μm (panels A, B, F, G, K, L, P, and Q) and 50 μm (panels C-E, H-J, M-O, and R-T), respectively.

**Figure S3. Distribution of *Nb-1*-expressing cells in the brain of a worker prepupa.**

*In situ* hybridization was performed using coronal sections of the brain of a single worker prepupa and an antisense (**A, E**) or sense riboprobe (**B, F**) for *Nb-1* RNA. Section 1 is anterior to Section 2. (**C, D and G, H**) The magnified views of squares surrounded with dotted lines in panels (**A and E**), respectively. The *Nb-1* RNA signals were indicated by red arrows. MB, mushroom body; α, α-lobe; OL, optic lobe; La, lamina; Me, medulla; Lo, lobula; AL, antennal lobe; Re, retina. Scale bars indicate 500 μm (**A, B, E, F**) and 100 μm (**C, D, G, H**).

**Figure S4. Distribution of *Nb-1*-expressing cells in the brain of a worker 1-day pupa (P1).**

*In situ* hybridization was performed using coronal sections of the brain of a single worker at the P1 stage and an antisense (**A, E, I**) or sense riboprobe (**B, F, J**) for *Nb-1* RNA. Sections 1-3 correspond to the anterior, middle, and posterior parts of a brain, respectively. (**C, D, and G, H, and K, L**) The magnified views of squares surrounded with dotted lines in panels (**A, E, I**), respectively. The *Nb-1* RNA signals were indicated by red arrows. MB, mushroom body; mCa, medial calyx; lCa, lateral calyx; α, α-lobe; β, β-lobe; OL, optic lobe; Me, medulla; Lo, lobula; AL, antennal lobe; Re, retina; SOG, subesophageal ganglion. Scale bars indicate 500 μm (**A, B, E, F, I, J**) and 100 μm (**C, D, G, H, K, L**).

**Figure S5. Distribution of *Nb-1*-expressing cells in the brain of a worker 2-day pupa (P2).**

(**A, F, K, P**) *In situ* hybridization was performed using coronal sections of the brain of a single worker at the P2 stage and an antisense riboprobe for *Nb-1* RNA. Section 1-4 correspond to the most anterior, anterior, posterior, and the most posterior parts of a brain. (**B-E, G-J, L-O, Q, R**) The magnified views of squares surrounded with dotted lines in panels (**A, F, K, P**), respectively. sections are shown. The *Nb-1* RNA signals were indicated by red arrows. MB, mushroom body; mCa, medial calyx; lCa, lateral calyx; α, α-lobe; β, β-lobe; OL, optic lobe; La, lamina; Me, medulla; Lo, lobula; AL, antennal lobe; Re, retina; SOG, subesophageal ganglion. Scale bars indicate 500 μm (**A, F, K, P**) and 100 μm (**B-E, G-J, L-O, Q, R**).

**Figure S6. Distribution of *Nb-1*-expressing cells in the brain of a worker 3-day pupa (P3).**

*In situ* hybridization was performed using coronal sections of the brain of a single worker at the P3 stage and an antisense (**A, F, K**) or sense riboprobe (**B, G, L**) for *Nb-1* RNA. Sections 1-3 correspond to the anterior, middle, and posterior parts of a brain, respectively. (**C-E, H-J, M-O**) The magnified views of squares surrounded with dotted lines in panels (**A, F, K**), respectively. The *Nb-1* RNA signals were indicated by red arrows. MB, mushroom body; mCa, medial calyx; lCa, lateral calyx; α, α-lobe; β, β-lobe; OL, optic lobe; Me, medulla; Lo, lobula; AL, antennal lobe; Re, retina; SOG, subesophageal ganglion. Scale bars indicate 500 μm (**A, B, E, F, I, J**) and 100 μm (**C, D, G, H, K, L**).

**Figure S7. Distribution of *Nb-1*-expressing cells in the brain of a worker 5-day pupa (P5).**

*In situ* hybridization was performed using coronal sections of the brain of a single worker at the P5 stage and an antisense (**A, F, K**) or sense riboprobe (**B, G, L**) for *Nb-1* RNA. Sections 1-3 correspond to the anterior, middle, and posterior parts of a brain, respectively. (**C-E, H-J, M-O**) The magnified views of squares surrounded with dotted lines in panels (**A, F, K**), respectively. The *Nb-1* RNA signals were indicated by red arrows. MB, mushroom body; mCa, medial calyx; lCa, lateral calyx; OL, optic lobe; La, lamina; Me, medulla; Lo, lobula; AL, antennal lobe; Re, retina; CC, central complex body; SOG, subesophageal ganglion. Scale bars indicate 500 μm (**A, B, F, G, K, L**) and 100 μm (**C-E, H-J, M-O**).

**Figure S8. Distribution of *Nb-1*-expressing cells in the brain of a worker 6-day pupa (P6).**

(**A, E, I, L**) *In situ* hybridization was performed using coronal sections of the brain of a single worker at the P6 stage and an antisense riboprobe for *Nb-1* RNA. Sections 1-4 correspond to the most anterior, anterior, posterior, and the most posterior parts of a brain, respectively. (**B-D, F-H, I-K, M**) The magnified views of squares surrounded with dotted lines in panels (**A, E, I, L**), respectively. The *Nb-1* RNA signals were indicated by red arrows. MB, mushroom body; mCa, medial calyx; lCa, lateral calyx; α, α-lobe; β, β-lobe; OL, optic lobe; La, lamina; Me, medulla; Lo, lobula; Re, retina; Oc, ocellus; AL, antennal lobe; CC, central complex body; SOG, subesophageal ganglion. Scale bars indicate 500 μm (**A, E, I, L**) and 100 μm (**B-D, F-H, J, K, M**).

**Figure S9. Distribution of *Nb-1*-expressing cells in the brain of a worker 7-day pupa (P7).**

(**A, E, I**) *In situ* hybridization was performed using coronal sections of the brain of a single worker at the P7 stage and an antisense riboprobe for *Nb-1* RNA. Sections 1-4 correspond to the most anterior, anterior, posterior, and the most posterior parts of a brain, respectively. (**B-D, F-H, J-L**) The magnified views of squares surrounded with dotted lines in panels (**A, E, I**), respectively. The *Nb-1* RNA signals were indicated by red arrows. MB, mushroom body; mCa, medial calyx; lCa, lateral calyx; α, α-lobe; β, β-lobe; OL, optic lobe; La, lamina; Me, medulla; Lo, lobula; Re, retina; Oc, ocellus; AL, antennal lobe; CC, central complex body; SOG, subesophageal ganglion. Scale bars indicate 500 μm (**A, E, I, L**) and 100 μm (**B-D, F-H, J-L**).

**Figure S10. Distribution of *Nb-1*-expressing cells in the brain of a worker 8-day (P8) pupa.**

*In situ* hybridization was performed using coronal sections of the brain of a single worker at the P5 stage and an antisense (**A, F, K**) or sense riboprobe (**B, G, L**) for *Nb-1* RNA. Sections 1-3 correspond to the anterior, middle, and posterior parts of a brain, respectively. (**C-E, H-J, M-O**) The magnified views of squares surrounded with dotted lines in panels (**A, F, K**), respectively. The *Nb-1* RNA signals were indicated by red arrows. MB, mushroom body; mCa, medial calyx; lCa, lateral calyx; α, α-lobe; β, β-lobe; OL, optic lobe; La, lamina; Me, medulla; Lo, lobula; Re, retina; AL, antennal lobe; CC, central complex body; SOG, subesophageal ganglion. Scale bars indicate 500 μm (**A, B, F, G, K, L**) and 100 μm (**C-E, H-J, M-O**).

**Figure S11. Changes in the distribution of *Nb-1*-expressing cells during MB development.**

*In situ* hybridization was performed using frontal coronal brain sections of workers at the larval (L5S and PP) and pupal stages (P1-P9) and a riboprobe for *Nb-1* RNA. Red arrowheads indicate areas for *Nb-1*-expressing cells located in the center of MB calyces. Red arrows indicate the other *Nb-1*-expressing cells. α, α-lobe; β, β-lobe. Scale bars indicate 100 μm.

**Figure S12. *Nb-1*-expression in the proliferating cells in the brain of worker PP.**

(**A, D**) Triple labeling by *in situ* hybridization of *Nb-1* RNA, BrdU immunohistochemistry, and nuclear staining with DAPI was performed using coronal brain sections of worker PP injected with BrdU. Section 1 is anterior to Section 2. (**Bi-Biii, Ci-Ciii and Ei-Eiii, Fi-Fiii**) Magnified views of the squares surrounded with white dotted lines (squares **B, C and E, F**) in panels (**A and D**), respectively. White arrows indicate *Nb-1*-expressing proliferating cells. MB, mushroom body; La, lamina; Me, medulla; Lo, lobula; Re, retina; AL, antennal lobe; CC, central complex body. Scale bars indicate 200 μm (**A, D**), 40 μm (**Bi-Biii, Ci-Ciii**) and 20 μm (**Ei-Eiii, Fi-Fiii**).

**Figure S13. *Nb-1*-expression in the proliferating cells in the brain of worker P1.**

Triple labeling by *in situ* hybridization of *Nb-1* RNA, BrdU immunohistochemistry, and nuclear staining with DAPI was performed using coronal brain sections of a single worker pupa at the P1 stage injected with BrdU. (**A**) Triple labeling using an antisense riboprobe for *Nb-1* RNA (magenta), anti-BrdU antibody (green), and DAPI (blue). The simultaneous DIC image is also shown (grey). White arrowheads show the areas for *Nb-1*-expressing MB cells. (**B**) Triple labeling using a sense riboprobe for *Nb-1* RNA (magenta), normal IgG (green), and DAPI (blue) as a negative control. (**Ci-Civ and Di-Div**) Magnified views of the squares surrounded with white dotted lines (squares **C and D**) in panel (**A**), respectively. The *Nb-1*-expressing proliferating cells are indicated with white arrowheads. The *Nb-1* expressing cells whose nuclei were not labeled by BrdU are indicated by cyan arrowheads. MB, mushroom body; La, lamina; Me, medulla; Lo, lobula; Re, retina; AL, antennal lobe; CC, central complex body. Scale bars indicate 200 μm (**A, B**) and 50 μm (**C, D**).

**Figure S14. *Nb-1* expression in the first-instar larva (L1).**

*In situ* hybridization was performed using coronal sections of a single female L1 larva and an antisense (**A**) or sense (**B**) riboprobes for *Nb-1*. Scale bars indicate 100 μm.

**Figure S15.** **Full-length fluorescence images of the blot and the gel for Fig. 4.**

(A) Full-length fluorescence image of the blot for the upper panel of Fig. 4(A). Samples are total RNAs extracted from the whole female (worker) and male (drone) embryos and the whole brains of pupae at the P5 stage. The top and bottom edges of the gel, the sample loaded line, and the bands for *Nb-1* were indicated with red arrows, a blue arrow, and an arrowhead, respectively, on the right. (B) Full-length fluorescence image of the gel stained with ethidium bromide for the lower panel of Fig. 4(A). The top and bottom edges of the gel, the sample loaded line, and the bands for 18S rRNA were indicated with red arrows, a blue arrow, and an arrowhead, respectively. (C) Full-length fluorescence image of the blot for the upper panel of Fig. 4(B). Samples are total RNAs extracted from the queen ovary and abdomen without ovary, and the whole female and male embryos. The top and bottom edges of the gel, the sample loaded line, and the bands for *Nb-1* were indicated with red arrows, a blue arrow, and an arrowhead, respectively, on the right. (D) Full-length fluorescence image of the gel stained with ethidium bromide for the lower panel of Fig. 4(B). The top and bottom edges of the gel, the sample loaded line, and the bands for 18S rRNA were indicated with red arrows, a blue arrow, and an arrowhead, respectively.

**Figure S16. *Nb-1* expression in the larval, pupal, and adult drone brains.**

Northern blotting was performed using total RNAs extracted from the brains of drones at the feeding fifth instar larval stage (L5F), prepual stage (PP), pupal stages (WE; white eye pupal stage corresponding to P1 of worker, BE; brown eye pupal stage corresponding to P3, DBE; dark brown eye pupal stage corresponding to P4, YB; yellow body pupal stage corresponding to P6, DGH; dark gray head pupal stage corresponding to P8, DBH; dark brown head pupal stage corresponding to P9) and adult stage. 5 and 4 individuals were used for L5F and PP samples, respectively, and 3 individuals were used for each of P1-P9 and adult samples. The bands for 18S rRNA bands were also detected by staining with ethidium bromide as loading controls (lower panel). The bands corresponding to *Nb-1* RNA and 18S rRNA are shown with black and white arrowheads, respectively, on the right.

**Figure S17.** **Full-length fluorescence images of the blot and the gel for Fig. S16.**

(A) Full-length fluorescence image of the blot for the upper panel of Fig. S16. Samples are total RNAs extracted from the brains of drones at the feeding fifth instar larval stage (L5F), prepual stage (PP), pupal stages (WE; white eye pupal stage corresponding to P1 of worker, BE; brown eye pupal stage corresponding to P3, DBE; dark brown eye pupal stage corresponding to P4, YB; yellow body pupal stage corresponding to P6, DGH; dark gray head pupal stage corresponding to P8, DBH; dark brown head pupal stage corresponding to P9) and adult stage. The top and bottom edges of the gel, the sample loaded line, and the bands for *Nb-1* were indicated with red arrows, a blue arrow, and an arrowhead, respectively, on the right. (B) Full-length fluorescence image of the gel stained with ethidium bromide for the lower panel of Fig. S16. The top and bottom edges of the gel, the sample loaded line, and the bands for 18S rRNA were indicated with red arrows, a blue arrow, and an arrowhead, respectively.

**Figure S18. Distribution of the *Nb-1*-expressing cells in the brain of drone pupa.**

(**A, E, I, J, M, N, Q, R**) *In situ* hybridization was performed using coronal sections of the brain of a single drone pupa and an antisense (**A, E, I, M, Q**) or sense (**J, N, R**) riboprobe for *Nb-1* RNA. Sections 1-5 correspond to the most anterior, anterior, middle, posterior, and the most posterior parts of a drone brain. (**B-D, F-H, K, L, and O, P, and S, T**) The magnified views of squares surrounded with dotted lines in panels (**A, E, I, M, Q**), respectively. MB, mushroom body; mCa, medial calyx; lCa, lateral calyx; α, α-lobe; OL, optic lobe; La, lamina; Me, medulla; Lo, lobula; Re, retina; AL, antennal lobe; CC, central complex body; SOG, subesophageal ganglion. Scale bars indicate 500 μm (**A, E, I, J, M, N, Q, R**) and 100 μm (**B-D, F-H, K, L, O, P, S, T**).
